# A multiple-oscillator mechanism underlies antigen-induced Ca^2+^ oscillations in Jurkat T-cells

**DOI:** 10.1016/j.jbc.2023.105310

**Published:** 2023-09-29

**Authors:** J. Cory Benson, Olivier Romito, Ahmed Emam Abdelnaby, Ping Xin, Trayambak Pathak, Sierra E. Weir, Vivien Kirk, Francisco Castaneda, Ryan E. Yoast, Scott M. Emrich, Priscilla W. Tang, David I. Yule, Nadine Hempel, Marie Potier-Cartereau, James Sneyd, Mohamed Trebak

**Affiliations:** 1 Department of Pharmacology and Chemical Biology, University of Pittsburgh School of Medicine, Pittsburgh, Pennsylvania, USA; 2 Vascular Medicine Institute, University of Pittsburgh School of Medicine, Pittsburgh, Pennsylvania, USA; 3 Graduate Program in Cellular and Molecular Physiology, The Pennsylvania State University College of Medicine, Hershey, Pennsylvania, USA; 4 Inserm UMR 1069, Nutrition Croissance Cancer, Faculté de Médecine, Université de Tours, Tours, France; 5 Department of Mathematics, University of Auckland, Auckland, New Zealand; 6 Division of Hematology/Oncology, Department of Medicine, University of Pittsburgh School of Medicine, Pittsburgh, Pennsylvania, USA; 7 UPMC Hillman Cancer Center, University of Pittsburgh School of Medicine, Pittsburgh, Pennsylvania, USA; 8 Department of Pharmacology and Physiology, University of Rochester, Rochester, New York, USA

**Keywords:** calcium, STIM1, STIM2, Orai, Jurkat, oscillations, IP3, CD3, NFAT1, CRAC, SOCE

## Abstract

T-cell receptor stimulation triggers cytosolic Ca^2+^ signaling by inositol-1,4,5-trisphosphate (IP_3_)-mediated Ca^2+^ release from the endoplasmic reticulum (ER) and Ca^2+^ entry through Ca^2+^ release-activated Ca^2+^ (CRAC) channels gated by ER-located stromal-interacting molecules (STIM1/2). Physiologically, cytosolic Ca^2+^ signaling manifests as regenerative Ca^2+^ oscillations, which are critical for nuclear factor of activated T-cells-mediated transcription. In most cells, Ca^2+^ oscillations are thought to originate from IP_3_ receptor-mediated Ca^2+^ release, with CRAC channels indirectly sustaining them through ER refilling. Here, experimental and computational evidence support a multiple-oscillator mechanism in Jurkat T-cells whereby both IP_3_ receptor and CRAC channel activities oscillate and directly fuel antigen-evoked Ca^2+^ oscillations, with the CRAC channel being the major contributor. KO of either STIM1 or STIM2 significantly reduces CRAC channel activity. As such, STIM1 and STIM2 synergize for optimal Ca^2+^ oscillations and activation of nuclear factor of activated T-cells 1 and are essential for ER refilling. The loss of both STIM proteins abrogates CRAC channel activity, drastically reduces ER Ca^2+^ content, severely hampers cell proliferation and enhances cell death. These results clarify the mechanism and the contribution of STIM proteins to Ca^2+^ oscillations in T-cells.

Calcium (Ca^2+^) is a ubiquitous second messenger that plays a central role in signal transduction (1, 2, 3, 4). In T lymphocytes, Ca^2+^ signals are critical in regulating differentiation, proliferation, activation, apoptosis, and cytokine production (1, 2, 5, 6, 7, 8). The activation of T-cells *via* stimulation of the T-cell receptors (TCR) causes increases in cytosolic Ca^2+^ levels (7, 8, 9, 10, 11). These TCR-induced rises in cytosolic Ca^2+^ often take the form of Ca^2+^ oscillations (7, 12, 13). Typically, in nonexcitable cells Ca^2+^ oscillations are initiated through the activation of receptor-linked phospholipase C (PLC), resulting in the hydrolysis of the plasma membrane (PM) phospholipid phosphatidylinositol-4,5bisposphate (PIP_2_) into inositol 1,4,5-trisphosphate (IP_3_) and diacylglycerol. IP_3_ subsequently activates IP_3_ receptors (IP_3_R) in the endoplasmic reticulum (ER) membrane, leading to the release of Ca^2+^ from the ER stores, which can then be pumped back into the ER by the sarcoplasmic/endoplasmic reticulum calcium ATPase (SERCA), leading to repeated cytosolic Ca^2+^ oscillations. However, to be sustained for long durations, these Ca^2+^ oscillations require the transport of extracellular Ca^2+^ into the cytoplasm by the store-operated calcium entry (SOCE) pathway, mediated by the Ca^2+^ release-activated Ca^2+^ (CRAC) channel (2, 8, 14, 15). In the absence of extracellular Ca^2+^, oscillations typically cease after 1 to 3 cycles. Intriguingly, it was proposed that Ca^2+^ oscillations induced by TCR stimulation in T-cells are not derived from the typical cycles of IP_3_R-mediated Ca^2+^ release and SERCA reuptake observed in other nonexcitable cells. Instead, Dolmetsch and Lewis showed that low concentrations of SERCA inhibitors (*e.g.* 15 nM thapsigargin) that only partially block SERCA activity elicit Ca^2+^ oscillations in T-cells with similar shape and amplitude to those activated by TCR stimulation (16). These oscillations are supported and sustained directly by the oscillatory activity of the CRAC channel (13, 16), likely reflecting the small size of ER Ca^2+^ stores in T-cells.

The CRAC channel is a two component Ca^2+^ entry mechanism comprised of the ER Ca^2+^ sensors stromal interacting molecule 1 and 2 (STIM1 and STIM2), and the PM located and highly Ca^2+^-selective Orai channels, of which there are three isoforms, Orai1, Orai2, and Orai3 (15, 17, 18, 19, 20, 21, 22, 23, 24). When the ER stores are depleted, Ca^2+^ is released from the low affinity Ca^2+^-binding EF-hand domain of STIM proteins, causing STIM activation, aggregation, and localization to the ER-PM junctions, where they physically trap and gate Orai channels (22, 25, 26). The SOCE pathway was presumed essential for the refilling of the ER Ca^2+^ stores. However, many cell lines and primary cell types with deletions of both STIM1 and STIM2, including T-cells and B-cells, have largely maintained ER Ca^2+^ release from stores (27, 28, 29, 30, 31). For instance, HEK293 cells generated through CRISPR/Cas9 KO lacking STIM1 and STIM2 or lacking all three Orai proteins showed inhibited acute ER Ca^2+^ refilling when stores were depleted with cyclopiazonic acid (CPA). However, when those cells were maintained in culture, their ER Ca^2+^ stores eventually refilled to near normal levels (28, 32, 33), suggesting that alternative and slow acting pathways were able to refill the stores of these cells.

It is well established that SOCE is critical for signaling to the nucleus through a diverse array of transcription factors, including nuclear factor of activated T-cells (NFAT) isoforms resulting in the activation of equally varied transcriptional and metabolic programs and cellular responses (5, 17, 34, 35, 36, 37, 38, 39, 40, 41, 42). In T-cells, SOCE is of particular importance, as it is the primary means by which these cells sustain Ca^2+^ signaling (8, 15, 17) and this is evidenced by patients with loss of function mutations in STIM1 and Orai1 who suffer severe immune deficiency and autoimmunity (43). Notably, the oscillatory Ca^2+^ signaling induced by TCR stimulation and supported by SOCE serves as the key driver for the translocation of the NFAT transcription factor family to the nucleus (8, 44, 45). The NFAT family of proteins, in turn, regulate the expression of genes that are crucial to T cell activation, proliferation, differentiation, cytokine secretion, and metabolism (17, 37, 46, 47, 48, 49, 50, 51, 52, 53).

Despite their similarities, STIM1 and STIM2 are known to trigger SOCE and regulate Ca^2+^ signaling in different ways. STIM2 responds to subtle depletion of ER Ca^2+^ stores while STIM1 responds to substantial store depletion (8, 54). In accordance with this, STIM2 was first thought to play the homeostatic function of maintaining ER Ca^2+^ levels under resting conditions with STIM1 being the primary driver of receptor-activated SOCE in the majority of cell types (25, 26, 54, 55). In many cell types including primary T-cells and HEK293 cells, STIM1 KO inhibited thapsigargin-activated SOCE by 85 to 90% while STIM2 contributed the remaining 10 to 15% of SOCE (25, 26, 28, 54, 55, 56, 57). However, this appears to be a simplistic view as recent studies in HEK293 cells showed that both STIM proteins are actively involved in SOCE at all levels of store depletion while concomitantly regulating the activity of IP_3_ receptors, thus tailoring the frequency of Ca^2+^ oscillations to match the strength of agonist stimulation (28, 58). Nevertheless, the specific contribution of each STIM isoform to Ca^2+^ oscillations and downstream signaling in hematopoietic cell lines remains unclear.

Here, we used CRISPR/Cas9 to generate clones of Jurkat T-cells with gene KO of STIM1 and STIM2 to determine the differential functions of these two proteins in regulating Ca^2+^ signaling induced by TCR stimulation. We show that unlike the case of other nonexcitable cells, KO of either STIM1 or STIM2 significantly impairs SOCE and CRAC currents triggered by store depletion, although the inhibitory effect of STIM1 KO on SOCE and CRAC currents is slightly more efficient. Using these STIM single and double knockout (DKO) cells, we demonstrate the existence of a multiple-oscillator mechanism in Jurkat T-cells where both the IP_3_ receptors and CRAC channel activities oscillate, and directly fuel antigen-evoked Ca^2+^ oscillations, with the CRAC channel being the major contributor to Ca^2+^ oscillations as proposed by Dolmetsch and Lewis (16). Computational studies generated a mathematical model of Ca^2+^ oscillations in T-cells that is in line with our experimental data and further predicts that, in addition to the oscillatory function of the IP_3_ receptors and CRAC channels, the concentration of IP_3_ also oscillates during antigen-evoked Ca^2+^ oscillations. We further show that STIM1 and STIM2 synergize to maintain optimal Ca^2+^ oscillations with high amplitude upon TCR stimulation. As such, KO of either STIM isoform significantly inhibits the translocation of NFAT1, and the KO of both proteins completely halts NFAT1 nuclear translocation in response to store depletion, suggesting that both STIM proteins synergize to support NFAT1 induction. Accordingly, we reveal that KO of both STIM proteins dramatically reduces cell proliferation and survival. Surprisingly, KO of both STIM1 and STIM2 was associated with significantly inhibited ER Ca^2+^ store refilling, suggesting that SOCE is the major pathway that refills ER Ca^2+^ stores in these cells. Together, these results refine the current model of Ca^2+^ oscillations in T-cells and indicate that both STIM proteins are essential for generating and sustaining Ca^2+^ oscillations in T-cells and ensuring efficient NFAT1 nuclear translocation, ER refilling, survival, and proliferation.

## Results

### STIM KO inhibits SOCE, CRAC currents, and ER Ca^2+^ store refilling in Jurkat cells

**Figure 1:**
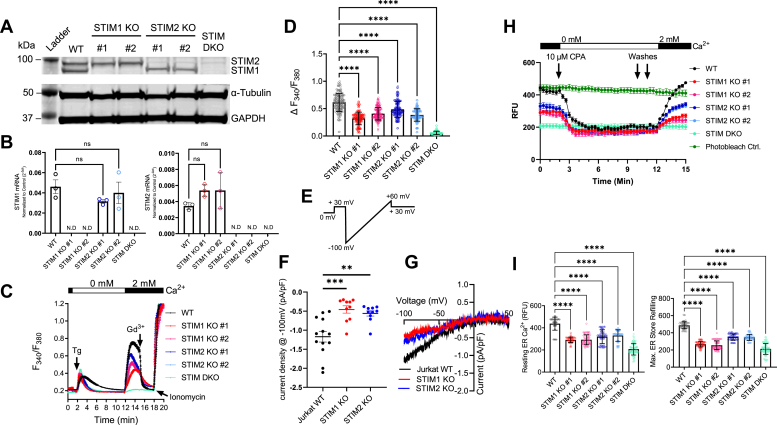
**STIM KO reduces CRAC currents, SOCE and ER refilling in Jurkat cells.***A*, representative Western blot confirming KO of STIM proteins in two independent clones for STIM1 and STIM2 each, and a pooled population of five STIM DKO clones. GAPDH and α–tubulin are used as housekeepers. *B*, qPCR confirming loss of STIM1 mRNA (*left*) and STIM2 mRNA (*right*). Cycle thresholds (CT) of STIM1 and STIM2 are normalized to the averaged CT of two housekeeper genes, GAPDH and α–tubulin. Error bars represent the SD. Results were analyzed by one-way ANOVA with post hoc Dunnett’s test (STIM1 F value = 16.62, *p* value <0.0001, STIM2 F value = 19.29, *p* value <0.0001). *C*, traces representing Fura-2 imaging to measure changes in cytosolic Ca^2+^ in Jurkat WT and STIM KOs. Store depletion is achieved with 2 μM thapsigargin added in Ca^2+^free HBSS and SOCE is measured by reintroduction of HBSS containing 2 mM Ca^2+^. SOCE is subsequently blocked by 5 μM Gd^3+^, and 10 μM Ionomycin is used as a loading control. All traces represent mean ± SEM of n = 180 cells per cell line. *D*, quantification of peak SOCE from (*C*), error bars represent the SD, results were analyzed by Kruskal-Wallis test with post-hoc Dunn’s multiple comparison (Kruskal-Wallis Statistic = 676.9). *E*, voltage ramp protocol used to elicit CRAC currents in Jurkat cells after dialysis with 10 mM EGTA through the patch pipette to deplete the ER stores. Results were analyzed by one-way ANOVA with post hoc Dunnett’s test (F value = 432.9, *p* value < 0.0001). *F*, mean ± SEM of CRAC current density at −100 mV in WT Jurkat cells, and their STIM1 KO and STIM2 KO counterparts. *G*, representative I/V curves of CRAC currents from WT, STIM1 KO, and STIM2 KO Jurkat cells. Results were analyzed by one-way ANOVA with post hoc Tukey's test (F value = 9.936, *p* value <0.001). *H*, traces representing CEPIAer imaging, which measures changes in ER Ca^2+^ store content in Jurkat WT and STIM KOs. Store depletion is achieved with reversible SERCA inhibition with 10 μM cyclopiazonic acid (CPA) added in Ca^2+^ free HBSS at t = 2 min (*arrow*), the cells are then washed twice with Ca^2+^-free HBSS a t = 10 min and t = 11 min to remove the CPA, and ER store refilling is measured by reintroduction of HBSS containing 2 mM Ca^2+^ at t = 12 min. All traces represent the mean ± SEM of n ≥ 67 cells per cell line. *I*, quantification of the average ER store Ca^2+^ levels at rest from time = 0 min to time = 1 min (*left*), and quantification of the maximum store refilling after reintroduction of 2 mM extracellular Ca^2+^ at t = 15 min (*right*). Statistical significance was determined by Kruskal–Wallis Test with post hoc Dunn’s multiple comparison test, where ∗∗∗∗ = *p* < 0.0001. (Resting ER Ca^2+^ Kruskal–Wallis Statistic = 276.9, *p* value <0.0001; Maximal ER Ca^2+^ Store Refilling Kruskal–Wallis Statistic = 353.7, *p* value <0.0001). All results are collected from at least three independent experiments. CRAC, Ca^2+^ release-activated Ca^2^; DKO, double knockout; ER, endoplasmic reticulum; HBSS, Hepes-buffered saline solution; qPCR, quantitative PCR; SERCA, sarcoplasmic/endoplasmic reticulum calcium ATPase; SOCE, store-operated calcium entry; STIM, stromal-interacting molecule.

**Figure 2:**
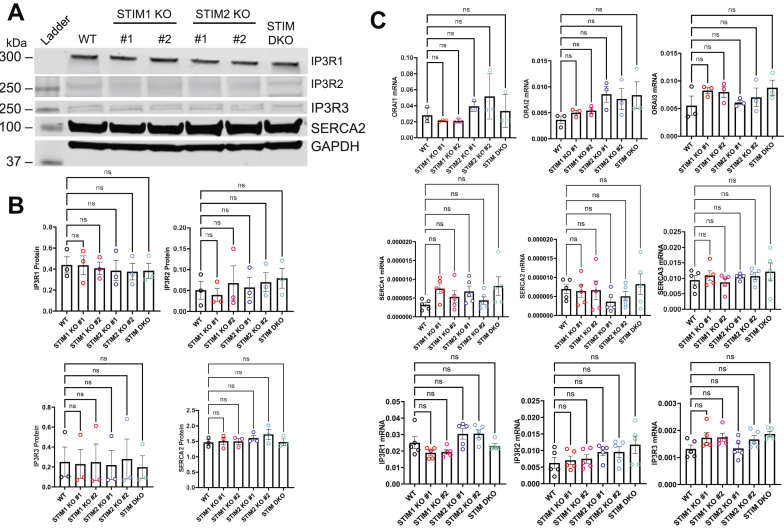
**STIM KO in Jurkat cells does not alter expression of IP**_**3**_**R, SERCA, and Orai.***A*, representative Western blot showing that KO of STIM1 or STIM2 does not affect the protein expression of the three types of IP_3_R or SERCA2. GAPDH is used as a control. *B*, quantification of protein expression from Western blots, proceeding from *left* to *right*: IP_3_R1, IP_3_R2, IP_3_R3, and SERCA2. Quantification was conducted by densitometry, and expression levels were normalized to GAPDH. Results were analyzed by one-way ANOVA with post hoc Dunnett's test (IP_3_R1 F value = 0.1230, *p* value = 0.9845; IP_3_R2 F value = 0.3079, *p* value = 0.8989; IP_3_R3 F value = 0.03238, *p* value = 0.9993; SERCA2 F value = 0.7671, *p* value 0.5909). *C*, (*top row*) qPCR showing that mRNA expression of Orai1 (*left*) Orai2 (*center*) and Orai3 (*right*) was not affected by KO of STIM proteins. (*Middle row*) qPCR indicating that mRNA expression of SERCA1 (*left*) SERCA2 (*center*) and SERCA3 (*right*) was not affected by KO of STIM proteins. (*Bottom row*) qPCR indicating that mRNA expression of IP_3_R1 (*left*) IP_3_R2 (*center*) and IP_3_R3 (*right*) was not affected by KO of STIM proteins. Cycle thresholds (CT) of each gene are normalized to the averaged CT of two housekeeper genes, GAPDH and α–tubulin. Statistical significance was determined by Kruskal–Wallis test with post hoc Dunn’s multiple comparison test, where ns = not significant. ORAI1 Kruskal–Wallis statistic = 9, *p* value = 0.1091; ORAI2 Kruskal–Wallis statistic = 10.26, *p* value = 0.0681; ORAI3 Kruskal–Wallis statistic = 5.117, *p* value = 0.4018; SERCA1 Kruskal–Wallis statistic = 8.066, *p* value = 0.1526; SERCA2 Kruskal–Wallis statistic = 3.699, *p* value = 0.5935; SERCA3 Kruskal–Wallis statistic = 2.590, *p* value = 0.7629; IP_3_R1 Kruskal–Wallis statistic = 14.83, *p* value = 0.0111; IP_3_R2 Kruskal–Wallis statistic = 8.448, *p* value = 0.1332; IP_3_R3 Kruskal–Wallis statistic = 8.406, *p* value = 0.1352. All results are collected from three independent experiments. IP_3_R, inositol-1,4,5-trisphosphate receptor; qPCR, quantitative PCR; SERCA, sarcoplasmic/endoplasmic reticulum calcium ATPase; STIM, stromal-interacting molecule.

To understand the differential role of the STIM isoforms in regulating Ca^2+^ signaling in T lymphocytes, we used Jurkat E6-1 cells as a suitable model to perform CRISPR/Cas9 single and double STIM1 and STIM2 gene KOs (Fig. 1). To mitigate any potential off-target effects from generating CRISPR/Cas9 KOs, two independent clones for the STIM1 and STIM2 KO were generated and studied. We also generated several clones of STIM1/STIM2 DKO. However, several independent attempts to grow these DKO clones resulted in the loss of all clones after two to three weeks of culture. Therefore, a polyclonal population comprised of five different STIM1/STIM2 DKO clones that although manifested very slow growth, have survived and was therefore used in our experiments. Clones were identified and validated for their respective KO by Sanger sequencing, Western blot, and quantitative PCR (qPCR) (Fig. 1, *A* and *B*). Genomic sequencing data of all our KO clones are included as a supplemental file. We also determined whether STIM KO resulted in any compensatory change in the Ca^2+^ handling proteins that are critical for store depletion, store refilling, and Ca^2+^ oscillations. We found no obvious compensatory changes in protein expression of the SERCA2 all three IP_3_R isoforms in all our STIM KO cell clones (Fig. 2, *A* and *B*). Similarly, we observed no changes in mRNA expression of the three Orai isoforms, the three SERCA isoforms, and the three IP_3_R isoforms across all STIM KO cells (Fig. 2C).

As T-cells are primarily dependent upon SOCE to propagate Ca^2+^-mediated signals, and SOCE is regulated by STIM1/2, we assessed the extent to which STIM isoform KO inhibited Ca^2+^ entry in response to store depletion by the SERCA blocker, thapsigargin (Tg; 2 μM; Fig. 1, *C* and *D*). Addition of thapsigargin to cells loaded with the Ca^2+^ dye Fura-2 in the absence of extracellular Ca^2+^ led to passive depletion of the ER stores. This ER Ca^2+^ store depletion triggers the activation of SOCE, which can be visualized upon the reintroduction of Ca^2+^ (2 mM) to the extracellular milieu. We then used the lanthanide gadolinium at a relatively low concentration (Gd^3+^; 5 μM) to selectively block Orai channels (24), confirming that the observed Ca^2+^ entry is mediated by STIM-activated CRAC channels. The ionophore ionomycin (10 μM) was added at the end of the recordings to ensure maximal Fura-2 signal across different cell lines. This protocol revealed a significant reduction in SOCE in the single STIM KO clones than the parental WT Jurkat line.

It is notable that KO of STIM1 nearly eliminates SOCE in a number of primary cell types and cell lines, including primary CD4+ T-cells (57). While STIM1 KO inhibited SOCE slightly more than STIM2 KO in Jurkat cells, STIM2 KO clones exhibited a significant reduction in SOCE (Fig. 1, *C* and *D*), suggesting that STIM2 has a more prominent role in mediating SOCE in Jurkat T-cells compared with other nonexcitable cells. This is similar to our previous studies in colorectal cancer cell lines showing that STIM2 has a large contribution to SOCE (56). Further, whole-cell patch clamp recordings in Jurkat T-cells showed that both STIM1 KO and STIM2 KO clones have significantly reduced CRAC currents triggered by dialysis of 10 mM EGTA through the patch pipette (Fig. 1, *F* and *G*) and elicited by voltage ramps from −100 mV to +60 mV (Fig. 1*E*). As expected, KO of both STIM1 and STIM2 resulted in the ablation of SOCE (Fig. 1, *C* and *D*), confirming that STIM1/2 are indeed the sole drivers of this pathway in Jurkat T-cells. Our repeated attempts to record CRAC currents from STIM1/2 DKO cells failed as these cells shrunk and died 1 to 2 min after contact with the recording pipette. These cells have low ER Ca^2+^ stores, exhibit increased cell death, and low proliferation rates and may likely have defects in membrane integrity and repair.

From the cytosolic Ca^2+^ recordings with Fura-2 above, it was readily apparent that the Ca^2+^ release phase, in the absence of extracellular Ca^2+^, was reduced in all STIM KO cells by comparison to the WT Jurkat cells (Fig. 1*C*), suggesting that the ER Ca^2+^ content is reduced in STIM KO cells. Therefore, we decided to perform direct measurements of ER Ca^2+^ using the genetically encoded ER-targeted dye CEPIAer (59). For these measurements, we used the reversible SERCA blocker CPA to deplete the stores in the absence of extracellular Ca^2+^, followed by CPA washout (2 washes as indicated by arrows in Fig. 1*H*) and subsequent reintroduction of 2 mM Ca^2+^ to the external milieu to determine the level of acute ER Ca^2+^ refilling (Fig. 1*H*). These experiments showed that the resting ER Ca^2+^ level and the level of acute ER refilling reached after store depletion with CPA were significantly lower in STIM KO cells compared to WT control cells with the following order of cell clones in basal ER Ca^2+^ content: STIM2KO>STIM1KO>STIM1/2 DKO (Fig. 1, *H* and *I*).

### STIM1 and STIM2 are essential for Ca^2+^ oscillations in Jurkat cells

**Figure 3:**
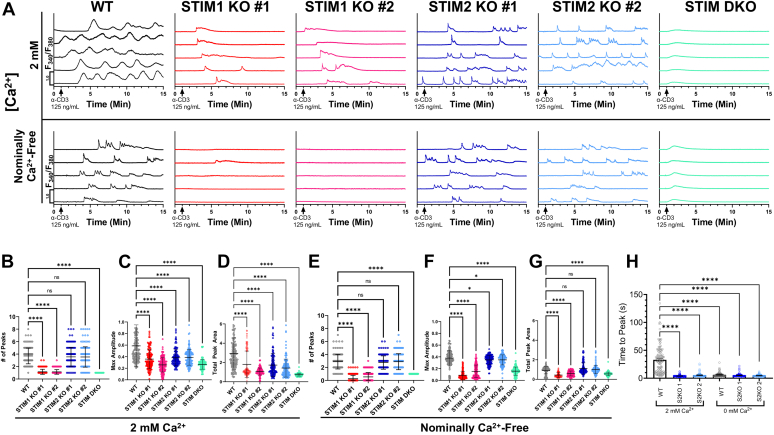
**STIM1and STIM2 KO alters Ca**^**2+**^**oscillations in Jurkat cells.***A*, representative traces of Fura-2 imaging of Ca^2+^ oscillations induced by 125 ng/ml α-CD3 antibody in the presence (*upper panels*) or absence (*lower panels*) of 2 mM extracellular Ca^2+^. *B*, quantification of the number of oscillation peaks in the presence of 2 mM Ca^2+^ in HBSS (n = 140 cells per cell line). Results were analyzed by one-way ANOVA with post hoc Dunnett's test (F value = 230.7, *p* value <0.0001). *C*, quantification of the maximum amplitude of oscillations from (*B*). Results were analyzed by one-way ANOVA with post hoc Dunnett's test (F value = 54.52, *p* value <0.0001). *D*, quantification of total oscillation peak area in oscillations from (*B*). Results analyzed by Kruskal–Wallis test with post hoc Dunn's test (Kruskal–Wallis statistic = 307.5, *p* value < 0.0001). *E*, quantification of the number of oscillation peaks in the absence of extracellular Ca^2+^ in HBSS (n = 140 cells per cell line). Results were analyzed by one-way ANOVA with post hoc Dunnett's test (F value = 286.7, *p* value <0.0001). *F*, quantification of the maximum amplitude observed in oscillations from (*E*). *G*, quantification of total oscillation peak area in oscillations from (*E*). Results analyzed by one-way ANOVA with post hoc’ Dunnett's test (F value = 265.4, *p* value < 0.0001). *H*, quantification of time-to-peak of oscillations from Jurkat WT and STIM2 KOs in the presence and absence of 2 mM Ca^2+^. Results were analyzed by Kruskal–Wallis test with post hoc Dunn’s multiple comparison test (Kruskal–Wallis statistic = 322.9). All results are collected from three independent experiments. Statistical significance for oscillation peak number and amplitude was determined by ordinary one-way ANOVA and corrected for multiple comparisons with Dunnett’s test, while oscillation period was analyzed by Kruskal–Wallis test and corrected for multiple comparisons with Dunn’s test, where ∗∗∗∗ = *p* <0.0001, ∗∗∗ = *p* <0.001, ∗∗ = *p* <0.01, ∗ = *p* <0.05, ns = not significant. HBSS, Hepes-buffered saline solution; STIM, stromal-interacting molecule.

TCR stimulation, typically simulated *in vitro* with anti-CD3 antibody, is a core mechanism by which T-cells are activated, and is known to produce cytosolic Ca^2+^ oscillations that drive NFAT translocation to the nucleus (10, 11, 60). To determine the specific contributions of STIM1 and STIM2 in regulating Ca^2+^ oscillations, we employed fluorescence microscopy using Fura-2 AM to monitor cytosolic Ca^2+^ levels in response to anti-CD3 stimulation (Fig. 3). In one set of experiments, Jurkat cells were kept in Hepes-buffered saline solution (HBSS) supplemented with 2 mM Ca^2+^ and stimulated with 125 ng/ml anti-CD3 antibody (α-CD3; Fig. 3*A*, top panels). Anti-CD3 stimulation produced a robust oscillatory response in the WT Jurkat cell line and in both STIM2 KO clones (Fig. 3, *A* and *B*). However, each Ca^2+^ oscillation in WT cells had bigger amplitude and period than oscillations in the STIM2 KO clones (Fig. 3, *C* and *D*), suggesting significant contribution to those oscillations in WT cells from STIM1-driven Ca^2+^ entry across the plasma membrane. By contrast, STIM1 KO clones showed mostly one initial oscillation (rarely two oscillations) while the STIM1/2 DKO clones showed almost no oscillations (at most one single low amplitude oscillation in some cells). These data suggest that both STIM proteins synergize for high amplitude optimal Ca^2+^ oscillations with STIM1 being the primary driver of TCR-induced Ca^2+^ oscillations in Jurkat T-cells (Fig. 3, *A*–*D*). These data also suggest that, unlike the case of other nonexcitable cells, in Jurkat cells SOCE has a major contribution to Ca^2+^ oscillations both directly and indirectly through refilling of ER Ca^2+^ stores.

In the second set of experiments, we recorded Ca^2+^ oscillations in an extracellular buffer lacking Ca^2+^ (Fig. 3*A*, bottom panels). The cells were kept in HBSS supplemented with 2 mM Ca^2+^ for the first minute of imaging. This external buffer was then replaced with Ca^2+^-free HBSS buffer prior to stimulation with α-CD3 where indicated by the arrow (Fig. 3*A*, bottom panels). In WT cells, we observed a reduction in Ca^2+^ oscillation frequency, amplitude, and period (Fig. 3, *E*–*G*), which reflected the absence of the contribution from Ca^2+^ entry across the plasma membrane through SOCE. Similar trends were observed in the STIM2 KO clones but with smaller oscillation amplitudes. Of note, when WT cells were stimulated in Ca^2+^ free HBSS, the oscillations were faster to reach peak and showed a bursting behavior (several oscillations on a raised plateau) compared to cells stimulated in 2 mM Ca^2+^ containing HBSS (compare traces in Fig. 3*A* and see quantification of times to reach peak in Fig. 3*H*). The pattern of oscillations of WT cells in Ca^2+^ free HBSS mimicked that of the STIM2 KO cells whether they were stimulated in Ca^2+^ free or in 2 mM Ca^2+^ (Fig. 3, *A* and *H*) solutions but with slightly smaller amplitude (Fig. 3*F*). These data suggest that the fast bursting oscillations in WT cells challenged with agonist in Ca^2+^ free HBSS are driven by IP_3_R-mediated Ca^2+^ release. The slightly reduced amplitude of these oscillations in STIM2 KO cells and their near absence in STIM1 KO and STIM1/2DKO cells (in either Ca^2+^ containing or Ca^2+^ free HBSS) reflect the relative contributions of STIM2 *versus* STIM1 to ER refilling.

However, the STIM1 KO clones and the STIM1/2 DKO cells had robust inhibition of Ca^2+^ responses (Fig. 3, *B*–*G*). The differences between WT cells and either STIM1 KO cells or STIM1/2 DKO cells observed in nominally Ca^2+^-free bath solutions are likely due to the defects in ER store refilling in STIM KO cells, as documented in Figure 1*H* and quantified in Figure 1*I*. The fact that we observed decreased maximum amplitude and period in the oscillations of all Jurkat lines in the absence of extracellular Ca^2+^ compared to the presence of extracellular Ca^2+^ strongly supports the idea that SOCE has a major and direct contribution to Ca^2+^ oscillations in Jurkat T-cells. If antigen-activated oscillations were driven entirely by SOCE, Ca^2+^ oscillations recorded in nominally Ca^2+^ free solutions would have been eliminated. Our data suggest that there is a contribution of intracellular ER Ca^2+^ stores in maintaining Ca^2+^ oscillations through IP_3_ receptor-mediated Ca^2+^ release, and that some of the Ca^2+^ entry through STIM1/2-dependent SOCE serves to acutely refill the ER Ca^2+^ stores during agonist stimulation.

### Orai channels are essential for Ca^2+^ oscillations

So far, we showed that the KO of both STIM proteins in Jurkat cells virtually eliminated Ca^2+^ oscillations in response to α-CD3 stimulation (Fig. 3). However, STIM1/2 KO also significantly reduced the levels of ER Ca^2+^ stores (Fig. 1). Therefore, to isolate the direct contribution of Ca^2+^ entry to Ca^2+^ oscillations from its indirect contribution to IP_3_-mediated Ca^2+^ release through ER store refilling, we sought to determine the impact of acute pharmacological inhibition of Orai channels on Ca^2+^ oscillations in the presence and absence of extracellular Ca^2+^.

**Figure 4:**
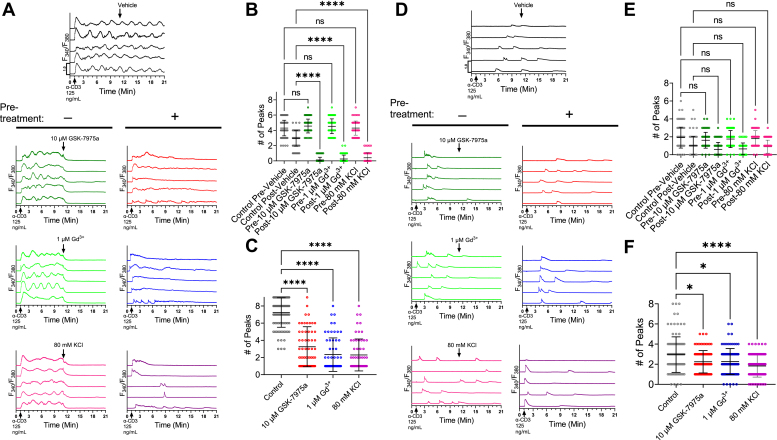
**Orai channel inhibition or membrane depolarization reduces Ca**^**2+**^**oscillations induced by α-CD3.***A*, representative traces of Fura-2 imaging in WT Jurkat cells stimulated with 125 ng/ml α-CD3 (at t =1 min) in the presence of 2 mM extracellular Ca^2+^ followed by addition of various inhibitors (*left panels*, at t= 11 min as indicated by *arrows*). In the *right panels*, cells were pretreated with either 10 μM GSK-7975a, 1 μM Gd^3^+ or 80 mM KCl before stimulation with 125 ng/ml α-CD3 at t = 1 min. *B*, quantification of oscillations observed in (*A*) without pretreatment (n ≥ 60 for each cell line). Kruskal–Wallis statistic = 680.8, *p* value <0.0001. Statistical significance was determined by Kruskal–Wallis Test with post hoc Dunn’s multiple comparison test, where ∗∗∗∗ = *p* < 0.0001, and where ns = not significant. *C*, quantification of oscillations observed in (*A*) with pretreatment (n ≥ 60 for each cell line). Kruskal–Wallis statistic = 146.6, *p* value < 0.0001. Statistical significance was determined by Kruskal–Wallis Test with post hoc Dunn’s multiple comparison test, where ∗∗∗∗ = *p* < 0.0001. *D*-*F*, similar experiments to those in (*A*–*C*), except that 2 mM extracellular Ca^2+^ was omitted. *E*, quantification of oscillations observed in (*D*) without pretreatment with inhibitors (n ≥ 60 for each cell line). Kruskal–Wallis statistic = 4.245, *p* value = 0.2362. Statistical significance was determined by Kruskal–Wallis Test with post hoc Dunn’s multiple comparison test where ns = not significant. *F*, quantification of oscillations observed in (*D*) with pretreatment with inhibitors (n ≥ 60 for each cell line). Kruskal–Wallis statistic = 24.62, *p* value < 0.0001. Statistical significance was determined by Kruskal–Wallis test with post hoc Dunn’s multiple comparison test, where ∗ = *p* <0.05 and where ∗∗∗∗ = *p* < 0.0001. All results are collected from three independent experiments.

First, WT Jurkat cells were stimulated with α-CD3 (125 ng/ml) in the presence of 2 mM extracellular Ca^2+^ and Orai channels were blocked with either low concentrations of Gd^3+^ (1 μM), or GSK-7975a (10 μM). In a third condition, we added 80 mM KCl to the external solution to induce plasma membrane depolarization and limit the driving force for Ca^2+^ entry through Orai channels (Fig. 4, *A*–*C*). These three Ca^2+^ entry inhibitors (Gd^3+^, GSK-7975a, and KCl) were either added simultaneously with α-CD3 antibody (at 1 min; Fig. 4*A*, right panels), or 10 min after addition of α-CD3 antibody where indicated by arrows (Fig. 4*A*, left panels). When we stimulated WT Jurkat cells with α-CD3 (125 ng/ml) and allowed the oscillations to proceed for 10 min, the subsequent addition of either Gd^3+^, GSK-7975a, or KCl, caused the oscillations to cease immediately (Fig. 4*A*, left panels and Fig. 4*B*). In all conditions of Jurkat cells stimulated with α-CD3 antibody and treated simultaneously with either Gd^3+^, GSK-7975a, or KCl (at 1 min), we observed a significant reduction in frequency of Ca^2+^ oscillations that was similar between the three inhibitors, although KCl and Gd^3+^ were slightly more effective than GSK-7975a (Fig. 4*C*). The remaining oscillations when Ca^2+^ entry was inhibited likely reflect contributions from ER Ca^2+^ stores, which eventually ran out in the absence of Orai-mediated Ca^2+^ entry (Fig. 4*A*, right panels). When SOCE inhibitors were added 10 min after stimulation with α-CD3 (Fig. 4*A*, left panels), Ca^2+^ oscillations ceases immediately in this case because ER Ca^2+^ stores were likely mostly depleted during the first 10 min of α-CD3 addition.

Second, we performed a similar set of experiments with the same protocols, stimuli and inhibitors, except that Ca^2+^ recordings were conducted in nominally Ca^2+^ free external solutions (Fig. 4, *D*–*F*). When Orai inhibitors or KCl were added at 10 min, the low amplitude and scarce Ca^2+^ oscillations proceeded largely unaffected by Orai inhibitors or membrane depolarization (Fig. 4*E*), although there was a small reduction in Ca^2+^ oscillation frequency in the presence of inhibitors that was not statistically significant. These results suggest that these oscillations in nominally Ca^2+^ free external solutions are driven strictly by ER Ca^2+^ release and are unaffected by membrane depolarization or Orai inhibitors. Surprisingly, when WT Jurkat cells stimulated with α-CD3 antibody in nominally Ca^2+^ free external solutions and treated simultaneously with either Gd^3+^, GSK-7975a, or KCl (at 1 min), we observed a slight but significant reduction in frequency of Ca^2+^ oscillations and this reduction was more pronounced with KCl than with Gd^3+^ or GSK-7975a (Fig. 4*F*). The simplest interpretation of these results is that prolonged exposure to nominally Ca^2+^ free solutions in combination with KCl or Orai blockers leads to more ER Ca^2+^ depletion (through Ca^2+^ leak) than exposure to nominally Ca^2+^ free alone.

### Mathematical modeling supports a multiple-oscillator mechanism in T-cells

**Figure 5:**
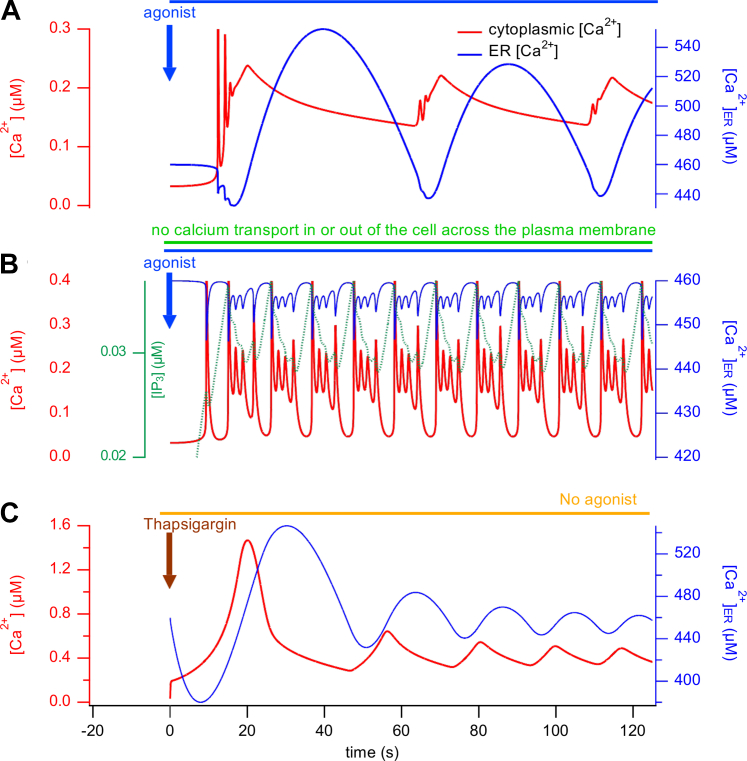
**Typical results of the mathematical model.** In the presence of extracellular Ca^2+^ allowing SOCE to occur, continuous application of agonist results in Ca^2+^ oscillations with a large amplitude and period (*A*, *red trace*). Also simulated are the changes in ER Ca^2+^ content with each oscillation (*A*, *blue trace*). When SOCE is removed from the model, the underlying IP_3_R-based oscillations of bursting form are revealed (*B*, *red trace*). Also simulated are the changes in ER Ca^2+^ content (*B*, *blue trace*) and IP_3_ levels (*B*, *green dashed trace*). In the simulation presented in (*B*), all movements of Ca^2+^, either into or out of the cell, are blocked, leading to oscillations that continue indefinitely. If influx is blocked but efflux is not, these bursting oscillations gradually run down and disappear. Simulation of the application of low concentrations of thapsigargin that only partially block SERCA, in the absence of agonist, gives low-frequency oscillations (*C*, *red trace*); also simulated ER Ca^2+^ levels (*C*, *blue trace*). ER, endoplasmic reticulum; IP_3_, inositol-1,4,5-trisphosphate; IP_3_R, IP_3_ receptor; SERCA, sarcoplasmic/endoplasmic reticulum calcium ATPase; SOCE, store-operated calcium entry.

Our experimental results suggest there are two concomitant and fundamentally different kinds of oscillations during TCR-mediated activation of Ca^2+^ signaling: IP_3_R-mediated and CRAC channel-mediated Ca^2+^ oscillations, and this conclusion is supported by a mathematical model that exhibits the same qualitative behaviors. When CRAC function is significant, large-period oscillations result from the interaction between ER depletion and CRAC channel activation (Fig. 5*A*, red trace); these oscillations depend critically on a time delay between these two events, as originally proposed by Dolmetsch and Lewis (16). When CRAC channel activity is decreased (through STIM1/2 KO or removal of extracellular Ca^2+^), the large-period oscillations are replaced by ones of qualitatively different shape, often taking the appearance of oscillations with bursts of faster oscillations on a raised plateau separated by quiescent periods (Fig. 5*B*). These bursts of faster oscillations on a raised plateau are clearly apparent in our experimental data when SOCE is inhibited through STIM KO or removal of extracellular Ca^2+^ (*e.g.* see Fig. 3*A*). In WT cells bathed in 2 mM extracellular Ca^2+^, the low-period oscillations are masked by the CRAC-based large-period oscillations, but are revealed when STIM1 or STIM2 are knocked out or when Ca^2+^ influx is reduced. However, passive depletion of the ER with low concentrations of thapsigargin (*e.g.* 15 nM) that partially block SERCA results in CRAC-based oscillations, in the absence of agonist stimulation (Fig. 5*C*) as experimentally documented by Dolmetsch and Lewis (16). Our mathematical model relies on four basic assumptions (1) SOCE has a steep dependence on ER Ca^2+^ concentration. (2) There is a time delay between ER depletion and SOCE. (3) IP_3_R can mediate faster oscillations in the same manner as seen in multiple other cell types (61). (4) A rise in Ca^2+^ increases the rate at which IP_3_ is degraded, resulting in periodic oscillations in the concentration of IP_3_ (61). Therefore, our mathematical model and our experimental data converge on three oscillators during TCR-evoked Ca^2+^ oscillations: (1) the large-period CRAC-based oscillations; (2) the intermediate-period IP_3_R-based oscillations mediated by oscillating IP_3_ concentrations; (3) within these IP_3_R-based oscillations reside bursts of faster oscillations on a raised plateau that are driven by cycles of cytosolic Ca^2+^-mediated activation and inhibition of IP_3_Rs.

It is somewhat counterintuitive that, although the agonist-induced oscillations in the presence of extracellular Ca^2+^ depend on ER depletion, the model predicts that the average ER concentration is slightly higher during these oscillations (Fig. 5*A*, blue trace) than it is during the oscillations in the absence of Ca^2+^ influx. This is because of the lag between changes in ER Ca^2+^ and changes in Ca^2+^ entry. Upon application of agonist in extracellular Ca^2+^ containing solution (Fig. 5*A*), an initial Ca^2+^ depletion from the ER (through the IP_3_R) activates a large CRAC current, but with a time delay. This causes a large increase in cytosolic Ca^2+^, which is then taken up into the ER by SERCA pumps. However, because of the time delay, this does not immediately shut down the CRAC current. Since the SERCA pumps are still working, the ER overfills. Eventually, CRAC switches off and the ER Ca^2+^ slowly decreases, leading to another delayed activation of CRAC, and the cycle repeats. A hint of the underlying process can be seen at the very beginning of the red trace in of Figure 5*A*. As the ER is depleting initially (immediately after application of the agonist), but before CRAC activity has had enough time to start, the model exhibits a short period of much faster oscillations (Fig. 5*A*; red trace), reminiscent of those seen in Fig. 5*B* that are dependent on Ca^2+^ modulation of the IP_3_R. However, in the red trace of Fig. 5*A*, those initial IP_3_R-based oscillations are quickly masked by the CRAC-based oscillations.

Even though they are initiated by activation of the IP_3_R, the CRAC-based oscillations are independent of any modulation of IP_3_R-mediated Ca^2+^ release; indeed, the model shows (computations not shown) that if the IP_3_R flux is replaced by a constant leak, SOCE-based oscillations still occur. In other words, although an oscillatory cytosolic Ca^2+^ will necessarily cause oscillatory Ca^2+^ release through the IP_3_R, such modulation of the IP_3_R is not required for CRAC-based oscillations to occur. It occurs simply as a byproduct of the properties of the IP_3_R.

When Ca^2+^ influx from outside through CRAC channels is minimized or prevented, a qualitatively different kind of oscillations can occur (Fig. 5*B*), taking the form of spikes with bursts of rapid oscillations on a raised plateau separated by quiescent periods. In contrast to CRAC-based oscillations, these bursting oscillations are absolutely dependent on the biphasic modulation of the IP_3_R by Ca^2+^. Furthermore, the IP_3_R-based oscillations and their bursting pattern rely also on an additional negative feedback mechanism, whereby an increase in cytosolic Ca^2+^ causes an increase in the degradation of IP_3_. In the model this occurs through the action of Ca^2+^ to enhance IP_3_-3 kinase activity that produces IP_4_ from IP_3_.

CRAC-based oscillations can also occur in the absence of agonist, but some mechanism is needed to provide an initial store depletion. As shown by (16), application of low concentrations of thapsigargin is sufficient to do this, and initiates CRAC-based oscillations, as seen in Figure 5*C*. During thapsigargin-induced oscillations, the SERCA pumps continue to work (but more slowly), thus allowing for refilling of the ER, assisted by the absence of any leak through the IP_3_R. Similarly, during thapsigargin-induced oscillations, the average ER concentration is slightly higher than at rest, as a result of the time delay between ER depletion and SOCE activation, as explained above.

### STIM isoforms synergize for NFAT1 nuclear translocation

**Figure 6:**
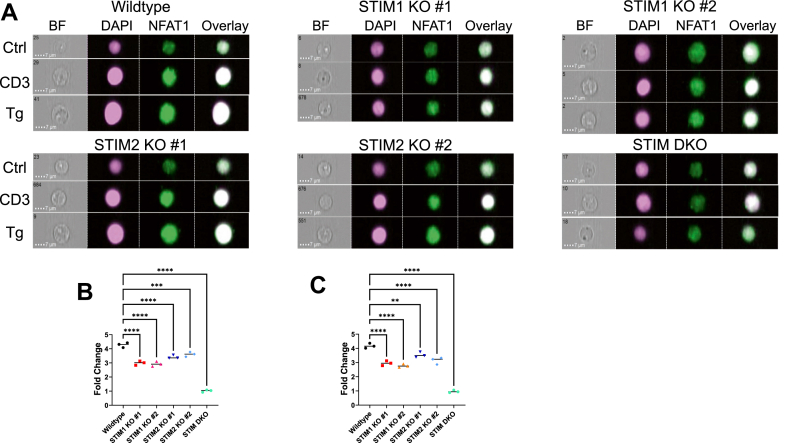
**KO of STIM proteins reduces nuclear translocation of NFAT1.***A*, representative images from Imagestream analysis of native NFAT1 translocation in untreated cells (Ctrl), and cells stimulated with either 125 ng/ml CD3 (CD3), or 2 μM thapsigargin (Tg) for 30 min. Images are shown with a 7 μm scale bar. NFAT1 was labeled with an NFAT1-specific primary antibody followed by a secondary antibody labeled with Alexa 647, and NFAT1 translocation was measured by colocalization with DAPI. *B*, quantification of the fold change of NFAT1 nuclear translocation from (*A*) comparing unstimulated Jurkat cells to Jurkat cells stimulated with thapsigargin. *C*, quantification of the fold change of NFAT1 nuclear translocation from (*A*) comparing unstimulated Jurkat cells to Jurkat cells stimulated with anti-CD3 antibody. The ImageStream experiment was conducted three independent times. Thapsigargin-stimulated Jurkat cells: F value = 119, *p* value < 0.0001; anti-CD3 antibody-stimulated Jurkat cells: F value = 162.7, *p* value <0.0001. Statistical significance was determined by one-way ANOVA corrected for multiple comparisons with Dunnett’s test where ∗∗∗∗ = *p* <0.0001, ∗∗∗ = *p* <0.001, ∗∗ = *p* <0.01. DAPI, 4′,6-diamidino-2-phenylindole; NFAT, nuclear factor of activated T-cells; STIM, stromal-interacting molecule.

A key role of the SOCE channel in T-cells is inducing the Ca^2+^-dependent activation of NFAT isoforms to promote proliferation, T-cell activation, and cytokine production (8, 62, 63, 64, 65). Among the five NFAT isoforms, NFAT1 is particularly prevalent in T-cells and is known to be activated by high cytosolic Ca^2+^ levels such as those induced by TCR activation (8, 66). Accordingly, we examined the effects of knocking out STIM1 and STIM2 isoforms on the nuclear translocation of native NFAT1 in Jurkat cells (Fig. 6). Jurkat cells were collected and either left untreated as a control or were treated with either thapsigargin (Tg; 2 μM) or α-CD3 antibody (125 ng/ml) in the presence of 2 mM extracellular Ca^2+^ for 30 min to activate SOCE. The cells were then fixed, permeabilized, and costained with an NFAT1 specific antibody and 4′,6-diamidino-2-phenylindole (DAPI). After staining the cells, the ImageStream multispectral imaging flow cytometer was used to capture NFAT1 translocation in tens of thousands of cells for each population (Fig. 6*A*). We discovered significantly reduced nuclear translocation of NFAT1 in STIM1 KO and STIM2 KO cells in response to both thapsigargin and α-CD3 stimulation, with STIM1 KO cells showing slightly more inhibition of NFAT1 nuclear translocation (Fig. 6, *B* and *C*). However, there was essentially no NFAT1 nuclear translocation in the STIM1/2 DKO cells in response to both thapsigargin and α-CD3 (Fig. 6, *B* and *C*).

### Loss of both STIM isoforms inhibits Jurkat proliferation and survival

**Figure 7:**
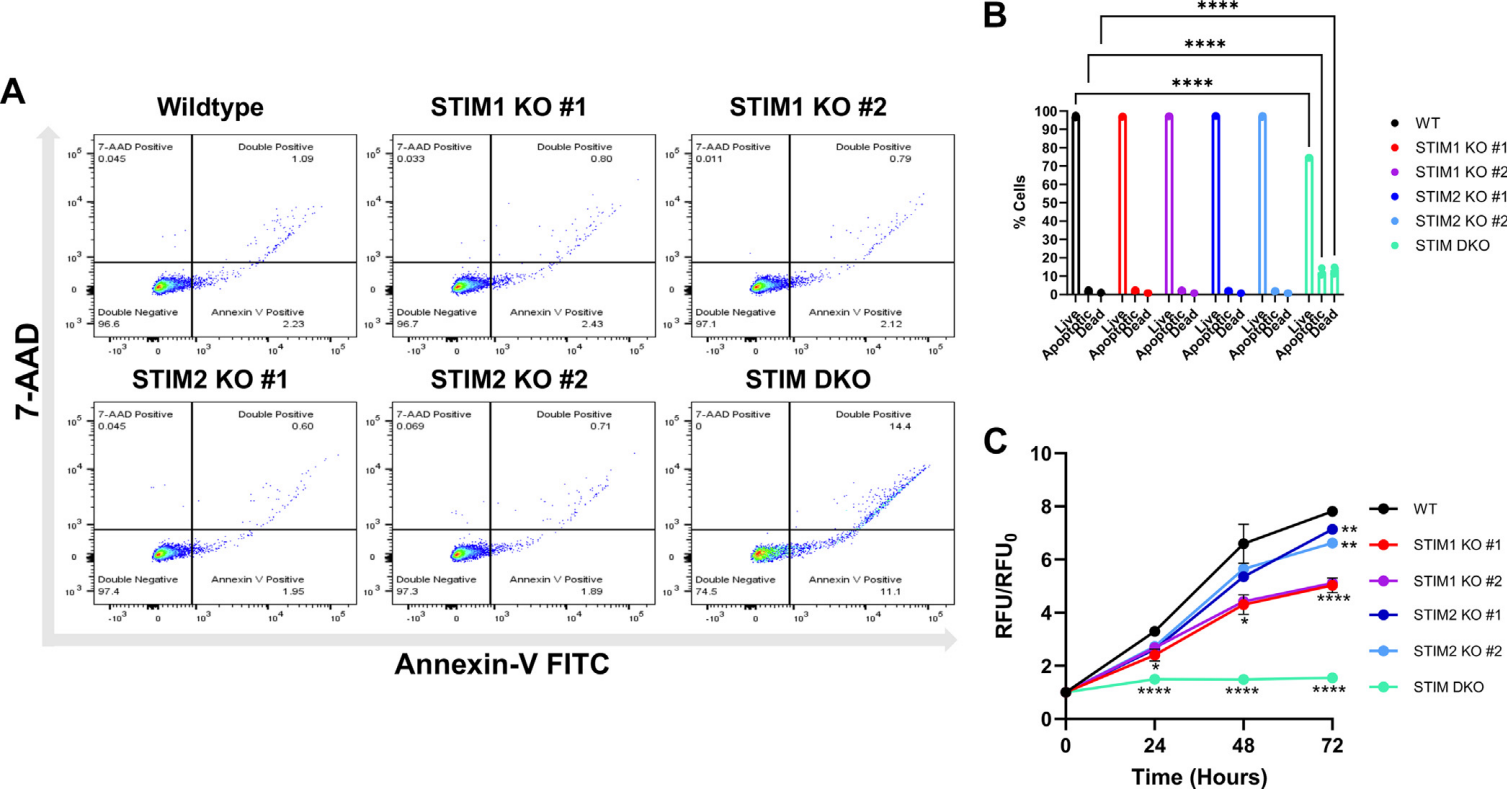
**Double KO of STIM proteins reduces Jurkat cell proliferation and enhances apoptosis.***A*, flow cytometry data of Annexin-V and 7-AAD labeled Jurkat cells. *Lower left quadrants* represent live cells, *lower right quadrants* represent cells in early apoptosis, *upper right quadrants* represent cells in late apoptosis or necrosis. *B*, quantification of flow cytometry data from (*A*). *C*, quantification of Jurkat cell proliferation in normalized relative fluorescence units (RFU) using the Cyquant proliferation assay. Flow cytometry interaction F value = 391.3, *p* value <0.0001; live/apoptotic/dead F value = 98,637, *p* value <0.0001; cell population F value = 0.05434, *p* value = 0.9980. Proliferation assay interaction F value = 104.5, *p* value <0.0001; time F value = 2,406, *p* value <0.0001; RFU/RFU_0_ F value = 2.431, *p* value = 0.0197. Data were derived from three independent experiments, statistical significance was determined by two-way ANOVA and corrected for multiple comparisons with Dunnett’s test. ∗∗∗∗ = *p* <0.0001, ∗∗∗ = *p* <0.001, ∗∗ = *p* <0.01, ns = not significant. AAD, amino-actinomycin D; STIM, stromal-interacting molecule.

As mentioned above, multiple attempts to generate and culture single clones of CRISPR/Cas9 STIM1/2 DKO cells in Jurkat cells were unsuccessful. While clones were selected in each attempt, we were unable to continuously culture them past two to three weeks as they failed to proliferate and eventually died out. During the fourth attempt to generate a stable population of STIM1/2 DKO cells, we observed a similar drop-off in colony survival after approximately 2 weeks. In an effort to prevent total loss of these new colonies, we reasoned that perhaps the low cell density in each population contributed to their failure to proliferate, and so we pooled the five surviving colonies into a single population to improve the odds of producing a stable population. This attempt proved successful, and we were able to maintain and use throughout this study the polyclonal STIM1/2 DKO population in cell culture, although this polyclonal population of STIM1/2 DKO cells grow significantly slower than the single STIM KO cells or their WT counterparts. To determine whether the consistent loss of STIM1/2 DKO clones was due to heightened apoptotic signaling or quiescence, we employed flow cytometry with Annexin V/7-amino-actinomycin D (7-AAD) co-staining and conducted a CyQUANT proliferation assay (Fig. 7). These assays revealed a significantly higher degree of early and late apoptotic cells among the STIM1/2 DKO cells (Fig. 7, *A* and *B*) than either the WT line or the single STIM KO clones. Indeed, we did not observe any difference between any single STIM KO clone and the WT line, suggesting that the cumulative effects of knocking out both STIM proteins are required to induce apoptosis. Similarly, the CyQUANT proliferation assay (Fig. 7*C*) revealed that the STIM DKOs display a heavily reduced proliferative rate in comparison to the WT cells and single STIM KOs. Nevertheless, the single STIM KO clones exhibited reduced proliferative rates in comparison to the parental WT line, with STIM1 KO cells displaying significantly more inhibition in proliferation than the STIM2 KO cells.

## Discussion

The common model of Ca^2+^ oscillations in most cells is initiated by release of Ca^2+^ from the ER stores by IP_3_R and sustained by influx of extracellular Ca^2+^ into the cytosol by SOCE and the reuptake of this cytosolic Ca^2+^ by SERCA (12, 67). However, previous studies showed that in T-cells low concentration of thapsigargin (*e.g.* 15 nM) that partially block SERCA can cause repetitive Ca^2+^ oscillations, which were noted to rely strictly on SOCE independently of IP_3_R activity (13, 16). While previous studies have examined the contributions of the STIM proteins to the maintenance of Ca^2+^ oscillations in several nonexcitable cell types, there have been diverging results reported, likely due in part to the use of siRNA to generate knockdowns of the proteins with varying degrees of efficiency (55, 68, 69, 70). As such, in our examination of Jurkat T-cells, we sought to avoid the confounding issues of incomplete knockdown by ensuring a clear genetic background in which only native STIM1 or STIM2 are expressed to assess their respective functions. Accordingly, we generated CRISPR/Cas9 STIM1 and STIM2 single KOs, and double STIM1/2 KO to determine their relative contributions to the generation and propagation of α-CD3-induced Ca^2+^ oscillations in Jurkat T-cells.

We found that the KO of either STIM1 or STIM2 results in significant reduction of SOCE, and that unlike primary T-cells and many other nonexcitable cell types, SOCE is not driven entirely by STIM1. The bigger contribution of STIM2 to SOCE might be a characteristic of some cancer cells as previous results showed larger contribution of STIM2 to SOCE in colorectal cancer cell lines (56). Nevertheless, we observed a near complete abrogation of Ca^2+^ oscillations with the KO of STIM1, while STIM2 KO cells were able to sustain oscillations, although these oscillations were of smaller amplitude highlighting the synergistic functions of STIM1 and STIM2 mediated SOCE in this process. Despite its inability to sustain oscillations on its own, STIM2 contributed to the amplitude of the Ca^2+^ oscillations, as the parental WT Jurkat cells generated Ca^2+^ oscillations with significantly greater amplitude than those of the STIM2 KO cells. This is in agreement with previous reports that found that STIM2 is involved in calcium signaling across the range of agonist concentrations and that it can recruit STIM1 to the ER–PM junctions under conditions of agonist stimulation that induce only modest ER store depletion (28, 69, 71, 72). The large amplitude of Ca^2+^ oscillations in WT Jurkat cells bathed in Ca^2+^ containing solutions, compared to those bathed in nominally Ca^2+^ free solutions, strongly suggest that SOCE plays a major role in Ca^2+^ oscillations in those cells across the time scale. Interestingly, however, we found that low amplitude oscillations were sustained in WT Jurkat and STIM2 KO cells in the absence of extracellular Ca^2+^, suggesting that in the absence of Ca^2+^ entry low amplitude and short-lived Ca^2+^ oscillations are mediated by IP_3_R-mediated Ca^2+^ release from the ER. This and our data showing that Ca^2+^ oscillations cease when Ca^2+^ entry is acutely inhibited by membrane depolarization or Orai channel blockers, argue that under physiological conditions of anti-CD3 stimulation, Ca^2+^ oscillations are dependent on a combination of ER Ca^2+^ release and concomitant Ca^2+^ entry. However, because the ER stores of these cells are relatively small, Ca^2+^ entry through STIM/Orai-mediated SOCE is the major contributor to Ca^2+^ oscillations in T-cells. Most likely, in other nonexcitable cells with larger ER Ca^2+^ stores such as HEK293 cells (28, 32), the direct contribution of oscillating CRAC channel activity, to each cycle of agonist-evoked Ca^2+^ oscillations, is significantly “drowned out” by IP_3_R-mediated ER Ca^2+^ release. Our results agree with previous studies concluding that Ca^2+^ oscillations induced by low concentrations of thapsigargin in T-cells are largely driven by SOCE (13, 16). Several studies have proposed that transient receptor potential canonical channels can be activated concomitantly with STIM/Orai upon receptor activation, thus contributing to Ca^2+^ oscillations and ER store refilling (73). However, the contribution of transient receptor potential canonical channels to Ca^2+^ signaling in primary T cells and Jurkat cells is uncertain (8).

Our experimental results and mathematical modeling support the existence of two concomitant and fundamentally different kinds of oscillations during TCR-mediated activation of Ca^2+^ signaling: IP_3_R-mediated and CRAC channel-mediated Ca^2+^ oscillations, with CRAC channels being major contributors to the bulk of cytosolic Ca^2+^ during oscillations. Our mathematical model suggest that these two concomitant types of TCR-evoked Ca^2+^ oscillations are mediated by three oscillators: (1) CRAC mediates the large period oscillations; (2) while the intermediate period IP_3_R-based oscillations are triggered by oscillating IP_3_ concentrations; (3) finally, within these IP_3_R-based oscillations reside bursts of faster oscillations on a raised plateau that are presumably driven by cycles of cytosolic Ca^2+^-mediated activation and inhibition of IP_3_Rs. We note that the data show that there must be a third oscillatory mechanism, not mediated by CRAC or by IP_3_R channels, operating on an intermediate time scale. Without this third oscillatory mechanism, it would be impossible to get fast oscillations occurring in bursts, with a quiescent period between the bursts. It is well established that feedback from rise of Ca^2+^ concentrations on the degradation of IP_3_ can cause such bursting behavior, and this is what our model assumes. This assumption can be considered a model prediction; there is, as yet, no direct evidence for this assumption, and yet it is necessary for the model to behave correctly.

KO of either STIM1 or STIM2 significantly inhibits NFAT1 translocation in response to store depletion by either thapsigargin or anti-CD3 antibody, in agreement with our previous findings in other cells (28). Here, it is notable that while loss of STIM1 resulted in greater inhibition of NFAT1 translocation than loss of STIM2, both STIM1 and STIM2 are capable of inducing similar degrees of NFAT1 translocation in Jurkat cells. This indicates an increased reliance on STIM2 in Jurkat cells than other cell types (25, 26, 28, 54, 55).

An unexpected outcome of particular interest in our study was the finding that KO of both STIM1 and STIM2 results in heavily impeded proliferation and survival of Jurkat T-cells. Indeed, the successful generation of a sustainable population of STIM1/2 DKO Jurkat cells was only achieved after four attempts. Functionally, the STIM1/2 DKO Jurkat cells displayed virtually no SOCE, no Ca^2+^ oscillations, low ER Ca^2+^ content, and no ability to induce the translocation of NFAT1 in response to thapsigargin or anti-CD3. Given that SOCE is the primary means through which T-cells sustain Ca^2+^ signals, and that Ca^2+^ signaling is central to numerous T-cell functions including proliferation, these results correspond well with our observation of increased cell death and inhibited proliferation in the STIM1/2 DKO cells. Curiously, however, this phenotype is not observed in primary CD4^+^ T-cells derived from KO mouse models. Instead, double STIM1/2 KO drastically affected the development and maturation of agonist-selected T-cells such as regulatory T-cells (57). This suggests that the phenotype observed in the Jurkat STIM1/2 DKO cells may be due to similarities between the Jurkat cell line and agonist-selected T-cells, or that it is specific to leukemic CD4^+^ T-cells or other cancerous cells. If the latter is true, this may offer a potentially exploitable target for the development of cancer therapeutics. However, this would require further exploration that is beyond the scope of the current work.

Taken together, our results indicate that STIM1 and STIM2 play differential and synergistic roles in the regulation of Ca^2+^ oscillations in Jurkat T-cells wherein STIM1 serves as the primary driver while STIM2 acts as an amplifier that facilitates the recruitment of STIM1 to ER–PM junctions. Under physiological conditions, Ca^2+^ oscillations in Jurkat T-cells are initiated by a combination of Ca^2+^ release from the ER and Ca^2+^ entry across the plasma membrane but rely heavily on STIM1/2-mediated SOCE for their long-term maintenance. Furthermore, our findings show that the loss of both STIM proteins has profound negative consequences on Ca^2+^ signaling, NFAT1 induction and proliferation of Jurkat cells that cannot be attributed to the loss of either protein individually. Thus, the synergistic contributions of both STIM1 and STIM2 are crucial for Ca^2+^ signaling, proliferation, and survival of Jurkat T-cells.

## Experimental procedures

The sequencing data of all CRISPR-Cas9 KO clones as well as the list of reagents and cell lines used in the study with their origin, identifier and additional information is provided in the supporting information file titled Reagents and Sequencing data. The raw data presented in the manuscript, including the original uncropped gels and Westerns are provided as supporting information data titled Source data for manuscript. All antibodies have been validated for their specificity by using KO and overexpression of the protein of interest in the relevant cell line.

### Cell culture

The parental Jurkat E6-1 cell line was purchased from the *American Type Culture Collection* (TIB-152). Cells were cultured in RPMI-1640 with L-Glutamine (Corning) supplemented with 10% heat-inactivated fetal bovine serum (Hyclone) and 1X penicillin-streptomycin (Corning). Cell lines were stored in a heated and humidified incubator under standard cell culture conditions (37 °C, 5% CO_2_, and 95% air). Cell lines were confirmed negative for *mycoplasma* contamination using a PCR detection kit (ABM: G238).

### Genetic KO of STIM1 and STIM2 in Jurkat cells using CRISPR/Cas9

To generate STIM KO in Jurkat cells, we employed CRISPR/Cas9 using lentiCRISPR v2 vectors we described previously (56). The Jurkat cells were transfected with the lentiCRISPR v2 vectors by electroporation using a Nucleofector II Device (Amaxa Biosystems) and subsequently incubated for 48 h under standard cell culture conditions. Clones were selected using the standard RPMI-1640 cell culture media supplement with puromycin (2 μg/ml; Gemini Bio Products) for 6 days. Once puromycin selection was complete, the clones were isolated by plating into 96-well plates at a density of one cell per well. KO of STIM1 and STIM2 were confirmed by Western blot analysis, Sanger sequencing, and Ca^2+^ imaging experiments.

### Western blot analysis

Cells were collected from culture flasks, pelleted *via* centrifugation, washed with ice-cold phosphate-buffered saline, and re-pelleted *via* centrifugation prior to lysis with ice-cold RIPA buffer (50 mM Tris pH 8.0, 150 mM NaCl, 0.5% sodium deoxycholate, 0.1% SDS, 1.0% IGEPAL; Millipore Sigma) supplemented with 1X Halt protease and phosphatase inhibitor (Thermo Fisher Scientific) for 20 min on ice. The crude protein lysates were clarified by centrifugation (15,000*g* for 10 min at 4 °C) and the supernatant was collected and total protein concentration was quantified by Pierce BCA assay (Thermo Fisher Scientific) according to the manufacturer’s protocols. Samples were then loaded onto precast NuPage 4% to 12% Bis–Tris gels, and the gels were run at 120V for 90 min. Proteins from completed gel runs were transferred to polyvinylidene difluoride (Millipore Sigma) membranes by wet transfer in Tris–glycine buffer (0.2 M Tris, 2M glycine, 20% methanol) overnight in a cold room (4 °C). Polyvinylidene difluoride membranes were then blocked with Intercept Blocking Buffer (LI-COR) for 1 h at room temperature (RT) before incubating with primary antibodies diluted in Intercept Blocking Buffer (1:10,000) for 1 h at RT. The membranes were washed three times with Tris-buffered saline with 0.1% Tween 20 (5 min per wash) and incubated with secondary antibodies (800CW-conjugated anti-rabbit (LI-COR) and 680RD-conjugated anti-mouse (LI-COR)) diluted (1:10,000) in Intercept Blocking Buffer for 1 h at RT. The membranes were then washed an additional three times as before, and blots were imaged used an Odyssey CLx Infrared Imaging System (LI-COR). GAPDH or α-tubulin was used as loading controls for all Western blots.

### Real-time quantitative reverse transcription PCR analysis

Total mRNA was collected from each cell line (5 × 10^6^ cells) using the RNeasy Mini Kit (Qiagen) according to the manufacturer’s protocols. Recovered RNA was measured using a Nanodrop 1000 Spectrophotometer (Thermo Fisher Scientific) and treated with DNAse I (Thermo Fisher Scientific) for 15 min at RT followed by treatment with 25 mM EDTA to terminate the digestion.1 μg of treated RNA was reverse transcribed using the High-Capacity cDNA Reverse Transcription Kit (Applied Biosystems) following manufacturer’s protocols. Total complementary DNA was diluted 1:10 in nuclease-free water (Bio-Rad), mixed with target-specific primers, and amplified with SYBR Green qPCR Master Mix (Applied Biosystems) at a final volume of 10 μl. All samples were amplified with the same PCR protocol beginning with a hot-start incubation for 2 min at 95 °C followed by 40 PCR cycles consisting of a 15 s denaturation step at 95 °C, a 15 s annealing step at 55 °C, and an extension step of 30 s at 72 °C. Subsequently, a standard melt curve was generated to ensure primer specificity and the comparative cycle threshold method was used to analyze target and control samples. Samples were normalized to two reference genes, GAPDH and α-tubulin. All samples were run with three technical replicates per qPCR plate, and three biological replicates were run to ensure reproducibility.

### Ca^2+^ imaging

Glass coverslips with a diameter of 25 mm were coated with poly-L-Lysine (0.01%; molecular weight 150–300 kDa; MilliporeSigma) 30 min prior to seeding. Jurkat cells were collected from cell culture flasks and pelleted by centrifugation (800*g* for 5 min) before being resuspended in HBSS containing 140 mM NaCl, 4.7 mM KCl, 1.13 mM MgCl_2_, 2.0 mM CaCl_2_, 10 mM glucose, and 10 mM Hepes with pH adjusted to 7.4 by NaOH. HBSS-suspended Jurkat cells were seeded at a density of 100,000 cells per coverslip. The Jurkat cells were allowed to adhere to the coverslips for a minimum of 15 min at RT to allow sufficient adhesion prior to mounting into an Attofluor cell chamber (Thermo Fisher Scientific). The mounted coverslips were incubated with 2 μM Fura-2-AM (Thermo Fisher Scientific) for 30 min at RT, and subsequently washed four times with HBSS to eliminate excess Fura-2. The chambers were then mounted on a Leica DMi8 Fluorescence Microscope. Cells were imaged using a 20X Fluor objective, and Fura-2 was excited at 340 and 380 nm using a fast shutter wheel (Sutter Instruments) with emissions captured at a wavelength of 510 nm with a Hamamatsu Flash 4 camera. The ratio of fluorescence at 340 and 380 nm (F_340_/F_380_) was analyzed using regions of interest drawn around the perimeters of 15 to 30 cells per coverslip using the Leica Application Suite X (LAS X) (https://www.leica-microsystems.com) software. The traces resulting from this analysis are represented as the mean ± SEM from at least three independent experiments.

### CyQUANT proliferation assay

Proliferation of the Jurkat cells was measured using the CyQUANT Cell Proliferation Assay (Thermo Fisher Scientific). A total of 2000 Jurkat cells from the WT line and each STIM KO population were seeded in complete media into separate wells of a 96-well tissue culture plate. For each recording, the 96-well plate was centrifuged for 5 min at 800*g* to pellet the cells, and then the wells to be read were washed with PBS and stained with CyQUANT dye for 1 h in a cell culture incubator. Fluorescence was read using a Synergy HT microplate reader (BioTek) using 490 nm excitation and 528 emission wavelengths. Background fluorescence from unstained wells was subtracted from each reading. The plate was returned to the cell culture incubator for overnight incubations between recordings. Recordings were captured immediately after seeding (0 h) and then at 24, 48, and 72 h. All recordings were normalized to their respective 0 h recording, and this ratio was represented as RFU/RFU_0_. Displayed results represent the means of three technical replicates per experiment collected from three independent experiments.

### Cell viability assay

To assess Jurkat apoptosis, cells were costained with FITC-Annexin V (Tonbo) and 7-AAD (Tonbo) and fluorescence was measured using flow cytometry. A total of 2 × 10^6^ Jurkat cells were collected per sample and pelleted by centrifugation prior to washing with ice cold PBS. The cells were then repelleted by centrifugation, and incubated with 100 μl of Annexin V binding buffer (150 mM NaCl, 2.5 mM CaCl_2_, 10 mM Hepes pH adjusted to 7.4 with NaOH) containing 5 μl of Annexin V and 5 μl of 7-AAD. The samples were incubated for 30 min at RT, and then diluted with 400 μl of Annexin V binding buffer prior to collecting data on a LSRFortessa (BD Biosciences). Data were generated from three independent experiments, and results were analyzed by FlowJo (https://www.flowjo.com/) software.

### Native NFAT1 translocation analysis with ImageStream

A total of 3 × 10^6^ Jurkat cells per condition were collected and transferred to 1.5 ml Eppendorf tubes. The cells were pelleted by centrifugation, and the media was replaced with 1X HBSS supplemented with 2 mM Ca^2+^. Cells were then treated for 30 min at RT with either thapsigargin (2 μM) to deplete the ER stores and induce SOCE or with 125 ng/ml α-CD3 to induce Ca^2+^ oscillations. After stimulation, the cells were fixed and permeabilized using the Foxp3/Transcription Factor Staining Kit following manufacturer protocols (Tonbo; TNB-0607-KIT). After fixation and permeabilization, the cells were centrifuged, the supernatant was removed, and the cells were incubated with an NFAT1 primary antibody at a 1:100 dilution (Cell Signaling Technology#4389S) for 1 h at RT. Unbound NFAT1 primary antibody was washed out, and the cells were stained using an Alexa-647-conjugated anti-rabbit secondary antibody (1:100; Invitrogen A21245) for 1 h at RT protected from light. Unbound secondary antibody was then washed out and the cells were resuspended in 40 μl of PBS. DAPI was used to stain the nucleus immediately before imaging by adding 10 μl of 1.25 μg/ml DAPI to the PBS (final concentration 0.25 μg/ml).

An Amnis ImageStream X Mk II Imaging Cytometer was used to capture NFAT1 translocation images. Bright field images were obtained with Channels 1 and 9 (430–480 nm), side scatter was monitored with Channel 6 (excitation wavelength 785 nm, emission wavelengths 745–800 nm), DAPI images were captured with Channel 7 (excitation wavelength 405 nm, emission wavelengths 430–505 nm), and Alexa-647 images were captured with Channel 11 (excitation wavelength 645, emission wavelengths 640–745 nm). Cells were gated to exclude doublets and debris and excitation laser power were adjusted to prevent pixel saturation before beginning image acquisition. Data were analyzed using IDEAS (https://www.emdmillipore.com/US/en/20150212_144049) software version 6.2 (Amnis Corporation) using the software’s transcription factor translocation guided analysis, identifying in-focus cells, excluding doublets, and ensuring cells were double-positive for DAPI and NFAT1. The raw nuclear similarity scores were exported to CSV files for statistical analysis. Displayed results represent similarity scores reported from three independent experiments.

### Patch clamp recordings

CRAC currents were measured using the whole cell configuration of the patch clamp technique on Jurkat WT, STIM1 KO, and STIM2 KO. Patch pipettes (2–4 MΩ) were pulled from borosilicate glass capillaries (World Precision Instruments) with P-1000 Flamming/Brown micropipette puller (Sutter Instrument Company). Data acquisition was performed with the Axopatch 200B, the Digidata 1440A (Molecular Devices) and was monitored with pCLAMP10 (https://www.moleculardevices.com/) software. Before recordings, cells suspended in HBSS were seeded on round coverslips pretreated with poly-L-Lysine for 10 min to allow cell adhesion. HBSS solution was washed with the bath solution. We selected cells with tight seals (>3 GΩ) and with <15 MΩ series resistance for recording. Cells were maintained at +30 mV during the experiments and were stimulated by a voltage ramp from −100 mV to +60 mV, lasting 250 ms every 3 s. Bath solution (mM): CaCl2, 20; NaCl, 130; KCl, 4.5; MgCl2, 1; D-glucose, 10; Hepes, 5 (pH 7.4 with NaOH). Pipette solution (mM): Cs methanosulfonate, 150; MgCl2, 8; EGTA, 10; Hepes, 10 (pH 7.2 with CsOH).

### The model equations

Let *c* denote [Ca^2+^]_i_, *c*_*e*_ denote [Ca^2+^]_ER_ and *p* denote [IP_3_]. Additionally, let *h* denote the activation rate of the IP_3_R by Ca^2+^, and let *j* be a variable which denotes the Ca^2+^ influx into the cell through the plasma membrane.

The model equations are:

(1) $$\begin{array}{c} \frac{dc}{dt}=J_{IPR}-J_{SERCA}+\delta \left(j-J_{PM}\right)\text{,} \end{array}$$

(2) $$\frac{dc_{e}}{dt}={\delta (J}_{SERCA}-J_{IPR})\text{,}$$

(3) $$\begin{array}{c} \tau_{h}\left(c\right)\frac{dh}{dt}=h_{\infty }\left(c\right)-h\text{,} \end{array}$$

(4) $$\begin{array}{c} \tau_{j}\frac{dj}{dt}=J_{in}-j\text{,} \end{array}$$

where

(5) $$\begin{array}{c} J_{\text{SERCA}}=\frac{V_{s}\left(c^{2}-\bar{K}c_{e}^{2}\right)}{c^{2}+K_{s}^{2}}\text{,} \end{array}$$

(6) $$\begin{array}{c} J_{\text{IPR}}=k_{f}P_{0}\left(c,p\right)\left(c_{e}-c\right)\text{,} \end{array}$$

(7) $$\begin{array}{c} J_{\text{PM}}=\frac{V_{p}c^{2}}{c^{2}+K_{p}^{2}}\text{,} \end{array}$$

(8) $$\begin{array}{c} h_{\infty }=\frac{K_{h}^{4}}{K_{h}^{4}+c^{4}}\text{,} \end{array}$$

(9) $$\begin{array}{c} J_{in}=\frac{V_{SOC}}{1+e^{s\left(c_{e}-K_{SOC}\right)}} \end{array}$$

Derivation of all these expressions can be found in (61). In a departure from earlier models, we assume that Ca^2+^ influx is a delayed response to ER depletion, instead of an instantaneous one. This delay is represented by the parameter $\tau_{j}$ and is the key to allowing the model to exhibit two different types of oscillations.

### Two types of oscillations

Ca^2+^ influx as a SOCE response is usually modeled as a Hill function with a relatively large coefficient to represent a low-affinity binding process that allows for the opening of the STIM-Orai channel. Here, we instead model SOCE as an exponential function, as seen in Equation 9, that, coupled with the delay $\tau_{j}$, creates a large Ca^2+^ entry that is able to “mask” IP_3_R-mediated Ca^2+^ release and allows the model to exhibit a large-amplitude, low-period oscillation. Oscillations that occur under conditions of minimal (or negligible) influx are the result of oscillatory Ca^2+^ fluxes through the IP_3_R. On the other hand, oscillations in WT cells have a higher amplitude and a larger period; these oscillations are caused primarily by transport of Ca^2+^ across the plasma membrane of the cell, and thus called SOCE oscillations.

### Bursting oscillations

The IP_3_R model used here is taken from (74) and is augmented by an additional equation for *p*, given by

$$\frac{dp}{dt}\;=\;V_{PLC}\;-\;\frac{V_{\deg}c^{2}}{c^{2}\;+\;{K_{\deg}}^{2}}p\text{.}$$

The first term represents production of IP_3_ by PLC and the second term represents decay of IP_3_ at a rate that is modulated by *c*. In this scenario, agonist stimulation of a cell is represented by an increase in the total production of PLC, given by $V_{PLC}$. Incorporation of negative feedback in this way allows for the appearance of bursting oscillations (Fig. 5*B*). Parameters of the model are listed in Table 1.

**Table 1:** Parameters of the model

SOCE		IP_3_ dynamics		IP_3_R		ATPase pumps	
τ_*j*_	80	*V*_deg_	0.3	*K*_*p*_	0.2	*K*_*s*_	0.2
*V*_soc_	25	*K*_deg_	0.2	*K*_*c*_	0.2	*V*_*s*_	0.9
*K*_soc_	250	τ_p_	1	*k*_β_	0.4	*K*_*p*_	0.25
*s*	1	*V*_PLC_	0.003 (0)	τ_max_	6	*V*_*p*_	0.2
γ	30			*K*_τ_	0.1	$\bar{K}$	1e-8 (4e-6)
δ	13			*k*_*f*_	1		
				*K*_*h*_	0.09		

All time units are in s, and all concentrations in mM. The parameters K_p_, K_c_, k_b_, K_t_ and t_max_ do not appear explicitly in the equations shown here, but are taken directly from Sneyd *et al.*, (2017). The values between parentheses are those used for the simulation of thapsigargin addition.

### Statistical analysis

All statistical analyses were conducted using GraphPad Prism 9 (https://www.graphpad.com/features). Data are presented as the mean ± SEM unless otherwise specified, and each point presented in scatter plots represents a single independent cell. All comparisons were analyzed using either one-way or two-way ANOVA, with parametric statistical tests used for normally distributed data, and nonparametric tests used when data were not normally distributed. GraphPad Prism 9 was employed to assess data normality (with D’Agostino and Pearson test, Anderson–Darling test, Shapiro–Wilk test, and Kolmogorov–Smirnov test). Statistical test results are displayed on each graph where ns, ∗, ∗∗, ∗∗∗, ∗∗∗∗ indicate *p*-values that are not significant, < 0.05, <0.01, <0.001, and <0.0001 respectively.

## Data availability

All raw data related to this manuscript are included as an online supplement.

## Supporting information

This article contains supporting information.

## Conflict of interest

Mohamed Trebak is a consultant for Seeker Biologics Inc, and is a member of the editorial board of the *J. Biol. Chem*. The remaining authors declare no competing interests.
