# Supplementary material for: A multiple-oscillator mechanism underlies antigen-induced Ca2+ oscillations in Jurkat T-cells
Source: J Biol Chem. 2023 Sep 29;299(11):105310. doi: 10.1016/j.jbc.2023.105310 (PMC10641176; doi:10.1016/j.jbc.2023.105310)
Supplement: Supporting Information 1 [file mmc1.docx]

**List of reagents and cells used in the study with their origin and identifier.**

| Reagent type (species) or resource | Designation | Source or reference | Identifiers | Additional information |
| --- | --- | --- | --- | --- |
| Antibody | Anti-GAPDH (Mouse monoclonal) | Millipore Sigma | Cat# MAB374, RRID:[AB_2107445](https://scicrunch.org/resolver/AB_2107445) | 1:10000 |
| Antibody | Anti-IP3R1 | Yule Lab | N/A |  |
| Antibody | Anti-IP3R2 | Yule Lab | N/A |  |
| Antibody | Anti-IP3R3 | Becton, Dickinson (BD) | Cat# 610313; RRID: AB_397705 | 1:1000 |
| Antibody | Anti-α-Tubulin (mouse monoclonal) | Cell Signaling Technologies | Cat# 3873S; RRID: AB_1904178 | 1:10000 |
| Antibody | Goat anti-rabbit Alexa-647-conjugated | Invitrogen | Cat# A21245 | 1:100 |
| Antibody | IRDye 800CW Donkey anti-Rabbit | Li-Core Biosciences | Cat# 925-32213 | 1:10000 |
| Antibody | IRDye 800CW Goat anti-Mouse | Li-Core Biosciences | Cat# 925-32210 | 1:10000 |
| Antibody | Mouse monoclonal anti-CD3E (Hit3a) | Tonbo Biosciences | Cat# 70-0039-U100 | 125 ng/mL |
| Antibody | Rabbit monoclonal anti-NFAT1 | Cell Signaling Technologies | Cat# 4389S | 1:100 |
| Antibody | Rabbit monoclonal anti-STIM1 | Cell Signaling Technologies | Cat# 5668; RRID: AB_10828699 | 1:10000 |
| Antibody | Rabbit polyclonal anti-SERCA2 | Cell Signaling Technologies | Cat# 4388S | 1:1000 |
| Antibody | Rabbit polyclonal anti-STIM2 | Cell Signaling Technologies | Cat# 4917; RRID: AB_2198021 | 1:10000 |
| Cell Line | Jurkat E6-1 | ATCC | TIB-152 |  |
| Cell Line | STIM1 KO Jurkat Cells | Trebak Lab | N/A |  |
| Cell Line | STIM1/2 KO Jurkat Cells | Trebak Lab | N/A |  |
| Cell Line | STIM2 KO Jurkat Cells | Trebak Lab | N/A |  |
| Chemical compound, drug | 7-AAD | Tonbo | SKU: 13-6993-T200 |  |
| Chemical compound, drug | CaCl_2_ | Fisher Scientific | Cat# C614 |  |
| Chemical compound, drug | Cesium hydroxide | Sigma-Aldrich | Cat# 232068-100G |  |
| Chemical compound, drug | Cesium Methanesulfonate | Sigma-Aldrich | Cat# C1426 |  |
| Chemical compound, drug | CRAC Channel Inhibitor IV, GSK-7975A | Sigma Aldrich | Cat# 5343510001 |  |
| Chemical compound, drug | Cyclopiazonic Acid | Alomone Labs | Cat# C-750 |  |
| Chemical compound, drug | DAPI | Sigma-Aldrich | Cat# D9542 | 1.25 µg/ml |
| Chemical compound, drug | Dextrose | Fisher Scientific | Cat# D14 |  |
| Chemical compound, drug | Dimethyl sulfoxide (DMSO) | Sigma | Cat# D2660-100ML | For solubilizing reagents, e.g. Fura-2-AM |
| Chemical compound, drug | EGTA | Fisher Scientific | Cat# O2783-100 |  |
| Chemical compound, drug | ExcelBand 3-color high range protein marker | Smobio | Cat# PM2600 |  |
| Chemical compound, drug | FITC Annexin V | Tonbo | SKU: 35-6409-T025 |  |
| Chemical compound, drug | Fura-2 AM | Thermo Fisher Scientific | Cat# F1221 |  |
| Chemical compound, drug | Gadolinium(III) Chloride | ACROS Organics | Cat# AC383560050 |  |
| Chemical compound, drug | Halt Protease and Phosphatase Inhibitor | Thermo Fisher Scientific | Cat# PI78443 |  |
| Chemical compound, drug | Heat-inactivated fetal bovine serum | Hyclone (Cytiva) | SH30071.03HI |  |
| Chemical compound, drug | HEPES | Fisher Scientific | Cat# BP310 |  |
| Chemical compound, drug | Intercept Blocking Buffer (TBS) | Li-Core Biosciences | Cat# 927-60001 |  |
| Chemical compound, drug | KCl | Fisher Scientific | Cat# P217 |  |
| Chemical compound, drug | LDS sample buffer | Thermo Fisher Scientific | Cat# NP0007 |  |
| Chemical compound, drug | MgCl_2_ | Fisher Scientific | Cat# M33 |  |
| Chemical compound, drug | MOPS SDS running buffer | Thermo Fisher Scientific | Cat# NP0001 |  |
| Chemical compound, drug | NaCl | Fisher Scientific | Cat# S671 |  |
| Chemical compound, drug | NuPAGE Bis-Tris precast gels | Thermo Fisher Scientific | Cat# NP0321 |  |
| Chemical compound, drug | Penicillin-Streptomycin | Corning | Cat# 3-002-CI |  |
| Chemical compound, drug | Poly-L-lysine solution | Sigma Aldrich | Cat# P4832-50ML |  |
| Chemical compound, drug | Polyvinylidene difluoride (PVDF) membrane | Li-Core Biosciences | Cat# 88518 |  |
| Chemical compound, drug | Precision plus protein dual color standards | Bio-Rad | Cat# 1610374 |  |
| Chemical compound, drug | Puromycin | Gemini Bio Products | Cat# 400128P |  |
| Chemical compound, drug | RPMI-1640 | Corning | Cat# 10-040CV |  |
| Chemical compound, drug | Thapsigargin | Thermo Fisher Scientific | Cat# T7458 |  |
| Chemical compound, drug | Tris Base | Fisher Scientific | Cat# BP152-5 |  |
| Chemical compound, drug | Tris-Glycine transfer buffer | Bio-rad | Cat#161-0734 |  |
| Chemical compound, drug | Tween 20 | Fisher Scientific | Cat# BP337 |  |
| Commercial assay or kit | BCA assay kit | Thermo Fisher Scientific | Cat# A53225 |  |
| Commercial assay or kit | cDNA Reverse Transcription Kit | Applied biosystems | Cat# 4368814 |  |
| Commercial assay or kit | Cell line nucleofector kit | Lonza | Cat# VCA-1003 |  |
| Commercial assay or kit | CyQUANT | Thermo Fisher Scientific | Cat# C35006 |  |
| Commercial assay or kit | DNase I Solution | Thermo Fisher Scientific | Cat# 89836 | Supplied with additional reagent buffer and EDTA |
| Commercial assay or kit | Foxp3/Transcription Factor Staining Kit | Tonbo | SKU: TNB-0607-KIT |  |
| Commercial assay or kit | Mycoplasma detection kit, PCR | ABM | G238 |  |
| Commercial assay or kit | RNeasy Mini Kit | Qiagen | Cat# 74104 |  |
| Commercial assay or kit | StrataClone Blunt PCR Cloning Kit | Agilent Technologies | Cat# 240207 |  |
| Commercial assay or kit | SYBER select master mix | Thermo Fisher Scientific | Cat# 4472920 |  |
| Recombinant DNA | LentiCRISPR v2 | Addgene | Cat# 52961 |  |
| Recombinant DNA | pCMV R-CEPIAer | Addgene | Cat# 58216 |  |
| Sequence-based reagent | GAPDH_F | Trebak Lab, this paper | RT-PCR primers | CCC TTC ATT GAC CTC AAC TAC A |
| Sequence-based reagent | GAPDH_R | Trebak Lab, this paper | RT-PCR primers | ATG ACA AGC TTC CCG TTC TC |
| Sequence-based reagent | IP3R1_F | Trebak Lab, this paper | RT-PCR primers | CAT TGC TGG GAA GCT AGA GAA G |
| Sequence-based reagent | IP3R1_R | Trebak Lab, this paper | RT-PCR primers | GTT CCA CCA GTG ACG AAG TAA A |
| Sequence-based reagent | IP3R2_F | Trebak Lab, this paper | RT-PCR primers | CAG CTC AGG CAG AAA CTA TGT |
| Sequence-based reagent | IP3R2_R | Trebak Lab, this paper | RT-PCR primers | CAG ATG AAT GAG GAC CCG TAA A |
| Sequence-based reagent | IP3R3_F | Trebak Lab, this paper | RT-PCR primers | GGG ATT ACA GAC TGC CTC TTC |
| Sequence-based reagent | IP3R3_R | Trebak Lab, this paper | RT-PCR primers | CTT CTC CTT GTC CTG CTT AGT C |
| Sequence-based reagent | ORAI1_F | Trebak Lab, this paper | RT-PCR primers | GAT GAG CCT CAA CGA GCA CT |
| Sequence-based reagent | ORAI1_R | Trebak Lab, this paper | RT-PCR primers | ATT GCC ACC ATG GCG AAG C |
| Sequence-based reagent | ORAI2_F | Trebak Lab, this paper | RT-PCR primers | TGG CGG AAG CTC TAC CTG AG |
| Sequence-based reagent | ORAI2_R | Trebak Lab, this paper | RT-PCR primers | CGG GTA CTG GTA CTG CGT C |
| Sequence-based reagent | ORAI3_F | Trebak Lab, this paper | RT-PCR primers | CTG GAG AGT GAC CAC GAG TA |
| Sequence-based reagent | ORAI3_R | Trebak Lab, this paper | RT-PCR primers | TGG AGA CCA TGA GTG CAA AG |
| Sequence-based reagent | Sequencing Primer STIM1 F | Trebak Lab, this paper | Sequencing Primer | TCA AGT GAG ACA TGT AAC AAA GAG G |
| Sequence-based Reagent | Sequencing Primer STIM1 R | Trebak Lab, this paper | Sequencing Primer | AAG AAA GGG GCT GCC TCA AG |
| Sequence-based reagent | Sequencing Primer STIM2 F | Trebak Lab, this paper | Sequencing Primer | ACT CCT GGT TGT AGT TGC CTG |
| Sequence-based reagent | Sequencing Primer STIM2 R | Trebak Lab, this paper | Sequencing Primer | TGA GAA ATA CAG GAA CAT GAG ATG G |
| Sequence-based reagent | SERCA1_F | Trebak Lab, this paper | RT-PCR primers | GTG ATC CGC CAG CTA ATG |
| Sequence-based reagent | SERCA1_R | Trebak Lab, this paper | RT-PCR primers | CGA ATG TCA GGT CCG TCT |
| Sequence-based reagent | SERCA2_F | Trebak Lab, this paper | RT-PCR primers | CGC TAC CTC ATC TCG TCC A |
| Sequence-based reagent | SERCA2_R | Trebak Lab, this paper | RT-PCR primers | TCG GGT ATG GGG ATT CAA |
| Sequence-based reagent | SERCA3_F | Trebak Lab, this paper | RT-PCR primers | GAT GGA GTG AAC GAC GCA |
| Sequence-based reagent | SERCA3_R | Trebak Lab, this paper | RT-PCR primers | CCA GGT ATC GGA AGA AGA G |
| Sequence-based reagent | STIM1 Guide 1 | Emrich et al. 2019 [https://doi.org/10.1074/jbc.RA118.006801](https://doi-org.ezaccess.libraries.psu.edu/10.1074/jbc.RA118.006801) | Guide RNA sequence | TGA TGA GCT TAT CCT CAC CA |
| Sequence-based reagent | STIM1_F | Trebak Lab, this paper | RT-PCR primers | ATG CCA ATG GTG ATG TGG AT |
| Sequence-based reagent | STIM1_R | Trebak Lab, this paper | RT-PCR primers | CCA TGG AAG GTG CTG TGT TT |
| Sequence-based reagent | STIM2 Guide 1 | Emrich et al. 2019 [https://doi.org/10.1074/jbc.RA118.006801](https://doi-org.ezaccess.libraries.psu.edu/10.1074/jbc.RA118.006801) | Guide RNA sequence | AGA TGG TGG AAT TGA AGT AG |
| Sequence-based reagent | STIM2 Guide 2 | Emrich et al. 2019 [https://doi.org/10.1074/jbc.RA118.006801](https://doi-org.ezaccess.libraries.psu.edu/10.1074/jbc.RA118.006801) | Guide RNA sequence | AGA AGA AGA CAG ATT TAG TC |
| Sequence-based reagent | STIM2_F | Trebak Lab, this paper | RT-PCR primers | CGC TGG CAA CAA ATT GAG AAG |
| Sequence-based reagent | STIM2_R | Trebak Lab, this paper | RT-PCR primers | CAC CCA GCT GTG ATC AGA ATA A |
| Sequence-based reagent | Tubulin_F | Trebak Lab, this paper | RT-PCR primers | AGT CCA AGC TGG AGT TCT CTA T |
| Sequence-based reagent | Tubulin_R | Trebak Lab, this paper | RT-PCR primers | CAA TCA GAG TGC TCC AGG GT |
| Software, algorithm | FlowJo 9.9.6 | <https://www.flowjo.com/solutions/flowjo> | RRID:[SCR_008520](https://identifiers.org/RRID/RRID:SCR_008520) |  |
| Software, algorithm | Graphpad Prism | <http://www.graphpad.com/> | RRID:[SCR_002798](https://identifiers.org/RRID/RRID:SCR_002798) |  |
| Software, algorithm | Image J | <https://imagej.net/> | RRID:[SCR_003070](https://scicrunch.org/resolver/SCR_003070) |  |
| Software, algorithm | Image Studio Lite | <https://www.licor.com/bio/image-studio-lite/download> | RRID:[SCR_013715](https://identifiers.org/RRID/RRID:SCR_013715) |  |
| Software, algorithm | Leica Application Suite X | <https://www.leica-microsystems.com/> | RRID:[SCR_016555](https://identifiers.org/RRID/RRID:SCR_016555) |  |

**Sequencing data for all CRISPR-Cas9 knockout Clones**

>Jurkat_Stim1_KO1_1-M13R_A01.ab1 **Insertion marked in red**
NNNNNNNNNNNNNNTACGCCAGCGCGCAATTAACCCTCACTAAAGGGAACAAAAGCTGGGTACCGGGCCCCCCCTCGAGGTCGACGGTATCGATAAGNNNGATATCCACTGTGGAATTCGCCCTTAAGAAAGGGGCTGCCTCAAGAGACCAGCAAGCTCCCTATCTCTGGAGATAAAGCAGCAGAACCTGGAAGATTCCTATGCCAGGGTTAGGTGGGTGAGGAAAGGGGTAGGAATAGGATAGAATTCAAAAAGTATCCTAATGAAGAAAAACGCCAACTCTGGAGAACCTTCCCCCATGCCCAGCAGAGTGACTATATTCAGTCTTTTTTAGTGACTATATGTAGAATAACTTCAATAATTTGCCCTTTACTAAGCATTTTATATACATGTTCTTCTAAGGCCAAGTTGCTGCTTCTCTCTTCTCTAAACCTTCCCACAACTAGGATTGATGACCAGGCTGCCTATTACCTTCTGATGACTTCCATGCCTTCCACAGGTCCTCCACGCTGATGAGCTTATCCTCA**C**CCATGGAAGGTGCTGTGTTTCACTGTTGGGTCATGGTAATTGAGGTCTTCCCTCAGGAACTGCAAGGGAAAAGTACACAAGTCACAAGAGATACCTAGGAGTCAGCCCGTCAAGATCTCCACACAGGTCACCTGCCTCTAGCTAGCCATAACACATTCCATCTACCCAACATACAAGTGTATCACATCACTCATGTATCCTTCCTTATGCACATGTCTTAGTTGCCAACTAAACTATCACTTCCTCTTTGTTACATGTCTCACTTGAAAGGCGAATTCCACATTGGTCGCTGCAGCCCGGGGGATCCACTAGTTCTAGAGCGGCCGCACCGCNNNGCTCCAATTCGCCCTATAGTGAGTCGTATTACGCGNNGCTCACTGGCCGTCGTTNACNACG

>Jurkat_Stim1_KO1_2-M13R_B01.ab1 **Mutation marked in blue, insertion marked in red**
NNNNNNNNNNNNNNNNCGCCAGCGCGCNATTAACCCTCACTAAAGGGAACAAAAGCTGGGTACCGGGCCCCCCCTCGAGGTCGACGGTATCGATAAGCTTGATATCCACTGTGGAATTCGCCCTTAAGAAAGGGGCTGCCTCAAGAGACCAGCAAGCTCCCTATCTCTGGAGATAAAGCAGCAGAACCTGGAAGATTCCTATGCCAGGGTTAGGTGGGTGAGGAAAGGGGTAGGAATAGGATAGAATTCAAAAAGTATCCTAATGAAGAAAAACGCCAACTCTGGAGAACCTTCCCCCATGCCCAGCAGAGTGACTATATTCAGTCTTTTTTAGTGACTATATGTAGAATAACTTCAATAATTTGCCCTTTACTAAGCATTTTATATACATGTTCTTCTAAGGCCAAGTTGCTGCTTCTCTCTTCTCTAAACCTTCCCACAACTAGGATTGATGACCAGGCTGCCTATTACCTTCTGATGACTTCCATGCCTTCCACAGGTCCTCCACGCTGATGAGCTTATCCTC**G**CCCN**G**GGAAGGTGCTGTGTTTCACTGNTGGGTCATGGTAATTGAGGTCTTCGCTCAGGAACNGCAAGGGAAAAGTACACNANTCACAACAGATACCTANGAGTCANCCNATCNAGATCTCCGCANNNGTCACCTGCCTCTAGCTAGCCATANCACACTCCATCTACCCAACATACAACTGTATCACATCGCTCATGTATCCNTCCTTATGCACNTGTCTTANTTGCCAACTAAACTATCACTTCCTCTTTGGTACNTGTCTCANTGGAAANGGCNAATTCNACATTGGTCGCTGCAGNNATGGGGATCCACTAGTTCTANAGCGGCCNNNNNGCNNNAGCTCCACTCNNCCTATACTGAGTCGTATTACNCNCGCTCACTGGNNNTCNTTTNNCANGNCGTGACNNN

>Jurkat_Stim1_KO1_3-M13R_C01.ab1 **5 nt deletion marked in red**
NNNNNNNNNNNNTTACGCCAGCGCGCANTTAACCCTCACTAAAGGGAACAAAAGCTGGGTACCGGGCCCCCCCTCGAGGTCGACGGTATCGATAAGCTTGATATCCACTGTGGAATTCGCCCTTTCAAGTGAGACATGTAACAAAGAGGAAGTGATAGTTTAGTTGGCAACTAAGACATGTGCATAAGGAAGGATACATGAGTGATGTGATACACTTGTATGTTGGGTAGATGGAATGTGTTATGGCTAGCTAGAGGCAGGTGACCTGTGTGGAGATCTTGACGGGCTGACTCCTAGGTATCTCTTGTGACTTGTGTACTTTTCCCTTGCAGTTCCTGAGGGAAGACCTCAATTACCATGACCCAACAGTGAAACACAGCACCTTCCATGGT **_ _ _ _ _** TAAGCTCATCAGCGTGGAGGACCTGTGGAAGGCATGGAAGTCATCANAAGGTAATAGGCAGCCTGGTCATCAATCCTAGTTGTGGGAAGGTTTAGAGAAGAGAGAAACAGCAACTTGGCCTTAGAAGAACATGTATATAAAATGCTTANTAAAGGGCAAATTATTGAAGTTATTCTACATATAGTCACTAAAAAAGACTGAATATAGTCACTCTGCTGGGCATGGGGGAAGGTTCTCCNNAGTTGGCGTTTTTCTTCATTANGATACTTTTTGAATTCTATCCTATTCCTACNCCTTTCCTCACCCACCTAACCCTGGCATAGGAATCTTCCANGTTCTGCTGCTTTATCTCCAGAGATAGGGAGCTTGCTGGTCTCTTGAGGCAGCCCCTTTCTTAANGGCGAATTCNACATTGGTCGCTGCAGCCCGGGGGATCCNCNNNNTCTAGAGCGGCCGCACCGCGNGAGCTCCAANTCNCCCTATAGTGAGTCGTATTACGCGCGCTCACTGNCCGTCGNTTTANNACGTCNNGACNGGGAAAACNCT

>Jurkat_Stim1_KO1_4-M13R_D01.ab1 **Insertion marked in red**
NNNNNNNNNCNNNNNTACGCCAAGCGCGCAATTAACCCTCACTAAAGGGAACAAAAGCTGGGTACCGGGCCCCCCCTCGAGGTCGACGGTATCGATAAGCTTGATATCCACTGTGGAATTCGCCCTTAGAAAGGGGCTGCCTCAAGAGACCAGCAAGCTCCCTATCTCTGGAGATAAAGCAGCAGAACCTGGAAGATTCCTATGCCAGGGTTAGGTGGGTGAGGAAAGGGGTAGGAATAGGATAGAATTCAAAAAGTATCCTAATGAAGAAAAACGCCAACTCTGGAGAACCTTCCCCCATGCCCAGCAGAGTGACTATATTCAGTCTTTTTTAGTGACTATATGTAGAATAACTTCAATAATTTGCCCTTTACTAAGCATTTTATATACATGTTCTTCTAAGGCCAAGTTGCTGCTTCTCTCTTCTCTAAACCTTCCCACAACTAGGATTGATGACCAGGCTGCCTATTACCTTCTGATGACTTCCATGCCTTCCACAGGTCCTCCACGCTGATGAGCTTATCCTCA**C**CCATGGAAGGTGCTGTGTTTCACTGTTGGGTCATGGTAATTGAGGTCTTCCCTCAGGAACTGCAAGGGAAAAGTACACAAGTCACAAGAGATACCTAGGAGTCAGCCCGTCAAGATCTCCACACAGGTCACCTGCCTCTAGCTAGCCATAACACATTCCATCTANNNACATACAAGTGTATCACATCACTCATGTATCCTTCCTTATGCACATGTCTTANTTGCCAACTAAACTATCACTTCCTCTTTGTTACATGTCTCACTTGAAAGGCGAATTCCACATTGGTCGCTGCAGCCCGGGGGATCCACTAGTTCTAGAGCGGCCGCACCGCNNAGCTCCAATTCGCCCTATAGTGAGTCGTATTACGCGCGCTCACTGGCCGTCNTTTTANANGTCNNGACTGGGNAAACCCTGGCGTTACCCAACTTAN

>Jurkat_Stim1_KO1_5-M13R_E01.ab1
**Vector only, result invalid**

>Jurkat_Stim1_KO1_6-M13R_F01.ab1 **5** **nt deletion marked in red**
NNNNNTNNNNNNNTTACGCCAGCGCGCANTTAACCCTCACTAAAGGGAACAAAAGCTGGGTACCGGGCCCCCCCTCGAGGTCGACGGTATCGATAAGCTTGATATCCACTGTGGAATTCGCCCTTAAGAAAGGGGCTGCCTCAAGAGACCAGCAAGCTCCCTATCTCTGGAGATAAAGCAGCAGAACCTGGAAGATTCCTATGCCAGGGTTAGGTGGGTGAGGAAAGGGGTAGGAATAGGATAGAATTCAAAAAGTATCCTAATGAAGAAAAACGCCAACTCTGGAGAACCTTCCCCCATGCCCAGCAGAGTGACTATATTCAGTCTTTTTTAGTGACTATATGTAGAATAACTTCAATAATTTGCCCTTTACTAAGCATTTTATATACATGTTCTTCTAAGGCCAAGTTGCTGCTTCTCTCTTCTCTAAACCTTCCCACAACTAGGATTGATGACCAGGCTGCCTATTACCTTCTGATGACTTCCATGCCTTCCACAGGTCCTCCACGCTGATGAGCTTA **_ _ _ _ _** ACCATGGAAGGTGCTGTGTTTCACTGTTGGGTCATGGTAATTGAGGTCTTCCCTCAGGAACTGCAAGGGAAAAGTACACAAGTCACAAGAGATACCTAGGAGTCAGCCCGTCAAGATCTCCACACAGGTCACCTGCCTCTAGCTAGCCATAACACATTCCATCTACCCAACATACAAGTGTATCACATCACTCATGTATCCTTCCTTATGCACATGTCTTAGTTGCCAACTAAACTATCACTTCCTCTTTGTTACATGTCTCACTTGAANGGCGAATTCCACATTGGTCGCTGCAGCCCGGGGGATCCACTANTTCTAGAGCGGCCGCACCGCGGGAGCTNCAATTCGCCCTATAGTGAGTCGTANTACGCGNNGCTCACTGGNCCGTCNTTTACNACGTCNNGACTGGGNAAACCNNNN

>Jurkat_Stim1_KO2_7-M13R_G01.ab1
NNNNNNNNNNNNTTACGCCAGCGCGCANTTAACCCTCACTAAAGGGAACAAAAGCTGGGTACCGGGCCCCCCCTCGAGGTCGACGGTATCGATAAGCTTGATATCCACTGTGGAATTCGCCCTTAAGAAAGGGGCTGCCTCAAGAGACCAGCAAGCTCCCTATCTCTGGAGATAAAGCAGCAGAACCTGGAAGATTCCTATGCCAGGGTTAGGTGGGTGAGGAAAGGGGTAGGAATAGGATAGAATTCAAAAAGTATCCTAATGAAGAAAAACGCCAACTCTGGAGAACCTTCCCCCATGCCCAGCAGAGTGACTATATTCAGTCTTTTTTAGTGACTATATGTAGAATAACTTCAATAATTTGCCCTTTACTAAGCATTTTATATACATGTTCTTCTAAGGCCAAGTTGCTGCTTCTCTCTTCTCTAAACCTTCCCACAACTAGGATTGATGACCAGGCTGCCTATTACCTTCTGATGACTTCCATGCCTTCCACAGGTCCTCCACGCTGATGAGCTTATCCTCACCCATGGAAGGTGCTGTGTTTCACTGTTGGGTCATGGTAATTGAGGTCTTCCCTCAGGAACTGCAAGGGAAAAGTACACAAGTCACAAGAGATACCTAGGAGTCAGCCCGTCAAGATCTCCACACAGGTCACCTGCCTCTAGCTAGCCATAACACATTCCATCTACCCAACATACAAGTGTATCACATNACTCATGTATCCTTCCTTATGCACATGTCTTAGTTGCCAACTAAACTATCACTTCCTCTTTGTTACATGTCTCACTTGAAAGGCGAATTCCACATTGGTCGCTGCAGCCCGGGGGATCCACTANTTCTAGAGCGGCCGCACCGCGGGAGCTCCANTTCGCCCTNNTANTGAGTCGTNNTACGCGCGCTCA

>Jurkat_Stim1_KO2_8-M13R_H01.ab1 **Insertion marked in red**
NNNNNNNNCNNNNTTACGCCNAGCGCGCAATTAACCCTCACTAAAGGGAACAAAAGCTGGGTACCGGGCCCCCCCTCGAGGTCGACGGTATCGATAAGCTTGATATCCACTGTGGAATTCGCCCTTAAGAAAGGGGCTGCCTCAAGAGACCAGCAAGCTCCCTATCTCTGGAGATAAAGCAGCAGAACCTGGAAGATTCCTATGCCAGGGTTAGGTGGGTGAGGAAAGGGGTAGGAATAGGATAGAATTCAAAAAGTATCCTAATGAAGAAAAACGCCAACTCTGGAGAACCTTCCCCCATGCCCAGCAGAGTGACTATATTCAGTCTTTTTTAGTGACTATATGTAGAATAACTTCAATAATTTGCCCTTTACTAAGCATTTTATATACATGTTCTTCTAAGGCCAAGTTGCTGCTTCTCTCTTCTCTAAACCTTCCCACAACTAGGATTGATGACCAGGCTGCCTATTACCTTCTGATGACTTCCATGCCTTCCACAGGTCCTCCACGCTGATGAGCTTATCCTCA**C**CCATGGAAGGTGCTGTGTTTCACTGTTGGGTCATGGTAATTGAGGTCTTCCCTCAGGAACTGCAAGGGAAAAGTACACAAGTCACAAGAGATACCTAGGAGTCAGCCCGTCAAGATCTCCACACAGGTCACCTGCCTCTAGCTAGCCATAACACATTCCATCTACCCAACATACAAGTGTATCACATCACTCATGTATCCTTCCTTATGCACATGTCTTAGTTGCCAACTAAANNANNACTTCCTCTTTGTTACATGTCTCACTTGAAAGGCGAATTCCACATTGGTCGCTGCAGCCCGGGGGATCCACTAGTTCTAGAGCGGCCGCACCGCGGGAGCTCCAATTCCGCCCTATANTGNGTCGTANTA

>Jurkat_Stim1_KO2_9-M13R_A02.ab1 **5 nt deletion marked in red**
NNNNNNNNNNNNNNTTACGCCAGCGCGCAATTAACCCTCACTAAAGGGAACAAAAGCTGGGTACCGGGCCCCCCCTCGAGGTCGACGGTATCGATAAGCTTGATATCCACTGTGGAATTCGCCCTTAAGAAAGGGGCTGCCTCAAGAGACCAGCAAGCTCCCTATCTCTGGAGATAAAGCAGCAGAACCTGGAAGATTCCTATGCCAGGGTTAGGTGGGTGAGGAAAGGGGTAGGAATAGGATAGAATTCAAAAAGTATCCTAATGAAGAAAAACGCCAACTCTGGAGAACCTTCCCCCATGCCCAGCAGAGTGACTATATTCAGTCTTTTTTAGTGACTATATGTAGAATAACTTCAATAATTTGCCCTTTACTAAGCATTTTATATACATGTTCTTCTAAGGCCAAGTTGCTGCTTCTCTCTTCTCTAAACCTTCCCACAACTAGGATTGATGACCAGGCTGCCTATTACCTTCTGATGACTTCCATGCCTTCCACAGGTCCTCCACGCTGATGAGCTTA **_ _ _ _ _** ACCATGNAANGTGCTGGGTNTCAGTGTTGGGNCAGGGTAATGGNNGTCTTCGTNNNCGAACTGGAACGGAAAAGTACACATATCANANTANATACATANGANTCAGACTCTCNAGATCTCCATACANGTCACCGGCANCTGNCTCGCNNTAACANTNTCAATCTACCCCACATNCANNTGTATCTGATCNNTCACGTACCCGTCNNNATGCNTATGTCNTAGTTGCANATTAAANTATCACTACCNCTTTGTCACNTGTCTCAGTTGAANNNGCNAAGTCNAATTCGNNCTCTGCCGCTCCGGNGATCNNGNNNNTCTAGATCGNNNGCNCCCCNGNGCTCGAANTCCCNCTATNCNGANNNNNATTCNNNNNCTCACTGNNNN

>Jurkat_Stim1_KO2_10-M13R_B02.ab1 **5 nt deletion marked in red**
NNNNNNNNNNNNNNNNCGCCAGCGCGCAATTAACCCTCACTAAAGGGAACAAAAGCTGGGTACCGGGCCCCCCCTCGAGGTCGACGGTATCGATAAGCTTGATATCCACTGTGGAATTCGCCCTTAAGAAAGGGGCTGCCTCAAGAGACCAGCAAGCTCCCTATCTCTGGAGATAAAGCAGCAGAACCTGGAAGATTCCTATGCCAGGGTTAGGTGGGTGAGGAAAGGGGTAGGAATAGGATAGAATTCAAAAAGTATCCTAATGAAGAAAAACGCCAACTCTGGAGAACCTTCCCCCATGCCCAGCAGAGTGACTATATTCAGTCTTTTTTAGTGACTATATGTAGAATAACTTCAATAATTTGCCCTTTACTAAGCATTTTATATACATGTTCTTCTAAGGCCAAGTTGCTGCTTCTCTCTTCTCTAAACCTTCCCACAACTAGGATTGATGACCAGGCTGCCTATTACCTTCTGATGACTTCCATGCCTTCCACAGGTCCTCCACGCTGATGAGCTTA **_ _ _ _ _** ACCATGGAANGNGCTGGGTTTCAGTGTTGGGTCAGGGTNANNGNNGTCNTCGTCCACGAACTGGAANGGAAAAGTACACNTATCNCAATANATACATANGANTCNGACTGTCCAGATCTCCATACANGTCANCGGCANCTGNCTAGCCATANCACNNTCNATCTACNNCACATNNAANTGTATCTGATCCCTCACGTACCCGTCNNTATGCATATGTCTTAGTTGNANNCTAAACTATCACTTCCTCTTCGTNACNTGTCTCAGTCGAAAGGGCNAAGTCNAAATTGNTCNCTGCCGCNCGGGGGATNGNGNNNTTCTAGATCGNNNGCNCCNNGNGAGCTCGAGCTCNCCTNNNNNGAGTCNGANTACNCNCGCNCACTNNNCNTCGNNNTNCNNCNTCANGACCGGGAAAGNNNNNNCGTTGNNN

>Jurkat_Stim1_KO2_11-M13R_C02.ab1 **5 nt deletion marked in red**
NNNNNNNNNNNNNTTACGCCAGCGCGCAATTAACCCTCACTAAAGGGAACAAAAGCTGGGTACCGGGCCCCCCCTCGAGGTCGACGGTATCGATAAGCTTGATATCCACTGTGGAATTCGCCCTTTCAAGTGAGACATGTAACAAAGAGGAAGTGATAGTTTAGTTGGCAACTAAGACATGTGCATAAGGAAGGATACATGAGTGATGTGATACACTTGTATGTTGGGTAGATGGAATGTGTTATGGCTAGCTAGAGGCAGGTGACCTGTGTGGAGATCTTGACGGGCTGACTCCTAGGTATCTCTTGTGACTTGTGTACTTTTCCCTTGCAGTTCCTGAGGGAAGACCTCAATTACCATGACCCAACAGTGAAACACAGCACCTTCCATGGT **_ _ _ _ _**TAAGCTCATCAGCGTGGAGGACCTGTGGAAGGCATGGAAGTCATCAGAAGGTAATAGGCAGCCTGGTCATCAATCCTAGTTGTGGGAAGGTTTAGAGAAGAGAGAAGCAGCAACTTGGCCTTAGAAGAACATGTATATAAAATGCTTAGTAAAGGGCAAATTATTGAAGTTATTCTACATATAGTCACTAAAAAAGACTGAATATAGTCACTCTGCTGGGCATGGGGGAAGGTTCTCCAGAGTTGGCGTTTTTCTTCATTAGGATACTTTTTGAATTCTATCCTATTCCTACCCCTTTCCTCACCCACCTAACCCTGGCATAGGAATCTTCCAGGTTCTGCTGCTTTATCTCCAGAGATAGGGAGCTTGCTGGTCTCTTGAGGCAGCCCCTTTCTTAAGGNGAATTCCACATTGGTCGCTGCAGCCCGGGGGATCCACTAGTTCTAGAGCGGCCGCACCGCGGGAGCTCCAATTCGCCCTATAGTGAGTCGTATTACGCGCGCTCACTGGNCCGTCGTTTTACNACGTCGTGACTGGGAANN

>Jurkat_Stim1_KO2_12-M13R_D02.ab1 **5 nt deletion marked in red**
NNNNNNNNNNNNNTTACGCCAGCGCGCANTTAACCCTCACTAAAGGGAACAAAAGCTGGGTACCGGGCCCCCCCTCGAGGTCGACGGTATCGATAAGCTTGATATCCACTGTGGAATTCGCCCTTAAGAAAGGGGCTGCCTCAAGAGACCAGCAAGCTCCCTATCTCTGGAGATAAAGCAGCAGAACCTGGAAGATTCCTATGCCAGGGTTAGGTGGGTGAGGAAAGGGGTAGGAATAGGATAGAATTCAAAAAGTATCCTAATGAAGAAAAACGCCAACTCTGGAGAACCTTCCCCCATGCCCAGCAGAGTGACTATATTCAGTCTTTTTTAGTGACTATATGTAGAATAACTTCAATAATTTGCCCTTTACTAAGCATTTTATATACATGTTCTTCTAAGGCCAAGTTGCTGCTTCTCTCTTCTCTAAACCTTCCCACAACTAGGATTGATGACCAGGCTGCCTATTACCTTCTGATGACTTCCATGCCTTCCACAGGTCCTCCACGCTGATGAGCTTA **_ _ _ _ _** ACCATGGAAGGTGCTGTGTTTCACTGTTGGGTCATGGTAATTGAGGTCTTCCCTCAGGAACTGCAAGGGAAAAGTACACAAGTCACAAGAGATACCTAGGAGTCAGCCCGTCAAGATCTCCACACAGGTCACCTGCCTCTAGCTAGCCATAACACATTCCATCTACCCAACATACAAGTGTATCACATCACTCATGTATCCTTCCTTATGCACATGTCTTAGTTGCCAACTAAACTATCACTTCCTCTTTGTTACATGTCTCACTTGAAAGGGCGAATTCCACATTGGTCGCTGCAGCCCGGGGGATCCACTAGTTCTAGAGCGGCCGCACCGCNNNGCTCCAATTCGCCCTATAGTGAGTCGTATTACGCGCGCTCACTGGCCGTCGTTTTACNACGTCGTGACTGGGAAAACCCTGGCGTTACCNAACTTAATCNNCNNN

>Jurkat_Stim2_KO1_13-M13R_E02.ab1 **62 nt insertion – 5 nt deletion region marked in blue**
NNNNNNNNCNNNNTTACGCCAGCGCGCAATTAACCCTCACTAAAGGGAACAAAAGCTGGGTACCGGGCCCCCCCTCGAGGTCGACGGTATCGATAAGCTTGATATCCACTGTGGAATTCGCCCTTTGAGAAATACAGGAACATGAGATGGAAAATTTTTATACAACTGGGAAGACTTATATCCAAATGGGGGGAAAAACAGAAAAACCATGTAAATGTTTATTAAGGGTAAAATTAACATGTAAAAAGAAAAATTAAAGATGTTGAAGATGAAGGCAATGAGCCACATGAGGAAAGAGAAAATTGAAGACCAGAAACAGAAATGCAGTCATTCTGTTCTAAGACAATAGCAAGGAAAACATTTTTCCACCTACTTCATCACTT**C**TCCTC**TCCTCTCTTTCTGTTTCTTTTTCCCTGTCTCTCTCTCTCTTTTTGTTTTACTGTAA**TACTTCAATTCCACCATCTTTGTCATCATCCATTTGTTTATGTATTGTTTGAAGAGCTTCCAGACTAAATCTGTCTTCTTCTGTAAAGCATGGTGGACTCAGTGACATGCAGGGATCTGAAAGAGAACAAAGGGCAATTACTAACTTGCCATACCAAAACAAAGGCTATAGTTAGAGAGTATAATTTAAAAGAAAACCAACAGAATTAAACTTCAGACTAAGTTAAACTAAGAGACTTTAAAACGTCTAACAGAATATTCTTCACATCAAAAGTATAATATATGAACACAACTGACTAAAAAGAAATAATCCAATTTAATTAACATCANGCAACTACAACCAGGAGTAAGGCGAATTCCACATTGGTCGCTGCAGCCCGGGGGATCCACTAGTTCTAGAGCGGCCGCACGCNNNGCTCCAATTCGCCCTATAGTGAGTCGTATTACGCGCGCTCACTGGCCGTCGTTTACNACGTCNTGACTGGNNAAACCCTGGNGTTACCCNNN

>Jurkat_Stim2_KO1_14-M13R_F02.ab1 **Same as Jurkat_STIM2_KO1_13-M13R_E02.ab1**
NNNNNNNNNNNNTTACGCCAGCGCGCAATTAACCCTCACTAAAGGGAACAAAAGCTGGGTACCGGGCCCCCCCTCGAGGTCGACGGTATCGATAAGCTTGATATCCACTGTGGAATTCGCCCTTACTCCTGGTTGTAGTTGCCTGATGTTAATTAAATTGGATTATTTCTTTTTAGTCAGTTGTGTTCATATATTATACTTTTGATGTGAAGAATATTCTGTTAGACGTTTTAAAGTCTCTTAGTTTAACTTAGTCTGAAGTTTAATTCTGTTGGTTTTCTTTTAAATTATACTCTCTAACTATAGCCTTTGTTTTGGTATGGCAAGTTAGTAATTGCCCTTTGTTCTCTTTCAGATCCCTGCATGTCACTGAGTCCACCATGCTTTACAGAAGAAGACAGATTTAGTCTGGAAGCTCTTCAAACAATACATAAACAAATGGATGATGACAAAGATGGTGGAATTGAAGTATTACAGTAAAACAAAAAGAGAGAGAGAGACAGGGAAAAAGAAACAGAAAGAGAGGAGAGGAGAAGTGATGAAGTAGGTGGAAAAATGTTTTCCTTGCTATTGTCTTAGAACAGAATGACTGCATTTCTGTTTCTGGTCTTCAATTTTCTCTTTCCTCATGTGGCTCATTGCCTTCATCTTCAACATCTTTAATTTTTCTTTTTACATGTTAATTTTACCCTTAATAAACATTTACATGGTTTTTCTGTTTTTCCCCCCATTTGGATATAAGTCTTCCCAGTTGTATAAAAATTTTCCATCTCATGTTCCTGTATTTCTCAAAGGGCGAATTCCACATTGGTCGCTGCAGCCCGGGGGATCCACTAGTTCTAGAGCGGCCGCNCNCGGGAGCTCCNNTTCGCCCTATAGTGAGTCGTATTACGCGCGCTCACTGGCNGTCGTTTTACNANGTCNTGACTGGNNNNN

>Jurkat_Stim2_KO1_15-M13R_G02.ab1 **Same as Jurkat_STIM2_KO1_13-M13R_E02.ab1**
NNNNNNNNNNNNNTTACGCCAGCGCGCAATTAACCCTCACTAAAGGGAACAAAAGCTGGGTACCGGGCCCCCCCTCGAGGTCGACGGTATCGATAAGCTTGATATCCACTGTGGAATTCGCCCTTTGAGAAATACAGGAACATGAGATGGAAAATTTTTATACAACTGGGAAGACTTATATCCAAATGGGGGGAAAAACAGAAAAACCATGTAAATGTTTATTAAGGGTAAAATTAACATGTAAAAAGAAAAATTAAAGATGTTGAAGATGAAGGCAATGAGCCACATGAGGAAAGAGAAAATTGAAGACCAGAAACAGAAATGCAGTCATTCTGTTCTAAGACAATAGCAAGGAAAACATTTTTCCACCTACTTCATCACTTCTCCTCTCCTCTCTTTCTGTTTCTTTTTCCCTGTCTCTCTCTCTCTTTTTGTTTTACTGTAATACTTCAATTCCACCATCTTTGTCATCATCCATTTGTTTATGTATTGTTTGAAGAGCTTCCAGACTAAATCTGTCTTCTTCTGTAAAGCATGGTGGACTCAGTGACATGCAGGGATCTGAAAGAGAACAAAGGGCAATTACTAACTTGCCATACCAAAACAAAGGCTATAGTTAGAGAGTATAATTTAAAAGAAAACCAACAGAATTAAACTTCAGACTAAGTTAAACTAAGAGACTTTAAAACGTCTAACAGAATATTCTTCACATCAAAAGTATAATATATGAACACAACTGACTAAAAAGAAATAATCCAATTTAATTAACATCNNCAACTACAACCAGGATAAGGCGAATTCCACATTGGTCGCTGCAGCCCGGGGGATCCACTAGTTCTAGAGCNNNCACGCGGGAGCTCNATTCGCCNATAGTGAGTCGTATTACGCGCGCTNACTGGCCGTCNTTTACNACGTNNNACTGGNNAANNCCTGGCGTTACCNNNN

>Jurkat_Stim2_KO1_16-M13R_H02.ab1 **Same as Jurkat_STIM2_KO1_13-M13R_E02.ab1**
NNNNNNNNNNNNNTTACGCCAGCGCGCATTAACCCTCACTAAAGGGAACAAAAGCTGGGTACCGGGCCCCCCCTCGAGGTCGACGGTATCGATAAGCTTGATATCCACTGTGGAATTCGCCCTTACTCCTGGTTGTAGTTGCCTGATGTTAATTAAATTGGATTATTTCTTTTTAGTCAGTTGTGTTCATATATTATACTTTTGATGTGAAGAATATTCTGTTAGACGTTTTAAAGTCTCTTAGTTTAACTTAGTCTGAAGTTTAATTCTGTTGGTTTTCTTTTAAATTATACTCTCTAACTATAGCCTTTGTTTTGGTATGGCAAGTTAGTAATTGCCCTTTGTTCTCTTTCAGATCCCTGCATGTCACTGAGTCCACCATGCTTTACAGAAGAAGACAGATTTAGTCTGGAAGCTCTTCAAACAATACATAAACAAATGGATGATGACAAAGATGGTGGAATTGAAGTATTACAGTAAAACAAAAAGAGAGAGAGAGACAGGGAAAAAGAAACAGAAAGAGAGGAGAGGAGAAGTGATGAAGTAGGTGGAAAAATGTTTTCCTTGCTATTGTCTTAGAACAGAATGACTGCATTTCTGTTTCTGGTCTTCAATTTTCTCTTTCCTCATGTGGCTCATTGCCTTCATCTTCAACATCTTTAATTTTTCTTTTTACATGTTAATTTTACCCTTAATAAACATTTACATGGTTTTTCTGTTTTTCCCCCCATTTGGATATAAGTCTTCCCAGTTGTATAAAAATTTTCCATCTCATGTTCCTGTATTTCTCAAAGGGCGAATTCCACATTGGTCGCTGCAGCCCGGGGGATCCACTAGTTCTAGAGCGGCCGCNCGCNNNGCTNCAATTCGCCCTATAGTGAGTCGTATTACNCGCGCTCACTGGCNGTCNTTTACNACGTCGTGACNGGGNAAANCCTGGCNN

>Jurkat_Stim2_KO1_17-M13R_A03.ab1 **Same as Jurkat_STIM2_KO1_13-M13R_E02.ab1**
NNNNNNNNNNNNNNNNACGCCAAGCGCGCANTTAACCCTCACTAAAGGGAACAAAAGCTGGGTACCGGGCCCCCCCTCGNGGTCGACGGTATCGATAAGCTTGATATCCACTGTGGAATTCGCCCTTTGAGAAATACAGGAACATGAGATGGAAAATTTTTATACAACTGGGAAGACTTATATCCAAATGGGGGGAAAAACAGAAAAACCATGTAAATGTTTATTAAGGGTAAAATTAACATGTAAAAAGAAAAATTAAAGATGTTGAAGATGAAGGCAATGAGCCACATGAGGAAAGAGAAAATTGAAGACCAGAAACAGAAATGCAGTCATTCTGTTCTAAGACAATAGCAAGGAAAACATTTTTCCACCTACTTCATCACTTCTCCTCTCCTCTCTTTCTGTTTCTTTTTCCCTGTCTCTCTCTCTCTTTTTGTTTTACTGTAATACTTCAATTCCACCATCTTTGTCATCATCCATTTGTTTATGTATTGTTTGAAGAGCTTCCAGACTAAATCTGTCTTCTTCTGTAAAGCATGGTGGACTCAGTGACATGCAGGGATCTGAAAGAGAACAAAGGGCAATTACTAACTTGCCATACCAAAACAAAGGCTATAGTTAGAGAGTATAATTTAAAAGAAAACCAACAGAATTAAACTTCAGACTAAGTTAAACTAAGAGACTTTAAAACGTCTAACAGAATATTCTTCACATCAAAAGTATAATATATGAACACAACTGACTAAAAAGAAATAATCCAATTTAATTAACATCANGCAACTACAACCAGGAGTANGGCGAATTCCACATTGGTCGCTGCAGCCCGGGGGATCCACTAGTTCTAGAGCGGCCGCACCGCGGGAGCTCCAATTCGCCCTATANTGAGTCGTATTACGCGCGCTCACTGGNCGTCGTTTTACNACGTCNTGACT

>Jurkat_Stim2_KO1_18-M13R_B03.ab1 **Same as Jurkat_STIM2_KO1_13-M13R_E02.ab1**
NNNNNNNNNCNTNNTTACGCCAGCGCGCAATTAACCCTCACTAAAGGGAACAAAAGCTGGGTACCGGGCCCCCCCTCGAGGTCGACGGTATCGATAAGCTTGATATCCACTGTGGAATTCGCCCTTTGAGAAATACAGGAACATGAGATGGAAAATTTTTATACAACTGGGAAGACTTATATCCAAATGGGGGGAAAAACAGAAAAACCATGTAAATGTTTATTAAGGGTAAAATTAACATGTAAAAAGAAAAATTAAAGATGTTGAAGATGAAGGCAATGAGCCACATGAGGAAAGAGAAAATTGAAGACCAGAAACAGAAATGCAGTCATTCTGTTCTAAGACAATAGCAAGGAAAACATTTTTCCACCTACTTCATCACTTCTCCTCTCCTCTCTTTCTGTTTCTTTTTCCCTGTCTCTCTCTCTCTTTTTGTTTTACTGTAATACTTCAATTCCACCATCTTTGTCATCATCCATTTGTTTATGTATTGTTTGAAGAGCTTCCAGACTAAATCTGTCTTCTTCTGTAAAGCATGGTGGACTCAGTGACATGCAGGGATCTGAAAGAGAACAAAGGGCAATTACTAACTTGCCATACCAAAACAAAGGCTATAGTTAGAGAGTATAATTTAAAAGAAAACCAACAGAATTAAACTTCAGACTAAGTTAAACTAAGAGACTTTAAAACGTCTAACAGAATATTCTTCACATCAAAAGTATAATATATGAACACAACTGACTAAAAAGAAATAATCCAATTTAATTAACATCANGCAACTACAACCAGGAGTNAAGGNGAATTCCACATTGGTCGCTGCAGCCCGGGGGATCCACTAGTTCTAGAGCGGCNCNCGCNNNGCTNCNNTTCGNCCNATAGTGAGTCGTATACNCGCGCTCACTGGNNTCGTTTANACGTCGTGACTGGGAAANCCTGNGTTACCNACTTANCNCTTGCAGCANATCCCCTTTCNCAGCNNN

>Jurkat_Stim2_KO1_19-M13R_C03.ab1 **Same as Jurkat_STIM2_KO1_13-M13R_E02.ab1**
NNNNNNNNNNNNNTTACGCCAGCGCGCAATTAACCCTCACTAAAGGGAACAAAAGCTGGGTACCGGGCCCCCCCTCGAGGTCGACGGTATCGATAAGCTTGATATCCACTGTGGAATTCGCCCTTACTCCTGGTTGTAGTTGCCTGATGTTAATTAAATTGGATTATTTCTTTTTAGTCAGTTGTGTTCATATATTATACTTTTGATGTGAAGAATATTCTGTTAGACGTTTTAAAGTCTCTTAGTTTAACTTAGTCTGAAGTTTAATTCTGTTGGTTTTCTTTTAAATTATACTCTCTAACTATAGCCTTTGTTTTGGTATGGCAAGTTAGTAATTGCCCTTTGTTCTCTTTCAGATCCCTGCATGTCACTGAGTCCACCATGCTTTACAGAAGAAGACAGATTTAGTCTGGAAGCTCTTCAAACAATACATAAACAAATGGATGATGACAAAGATGGTGGAATTGAAGTATTACAGTAAAACAAAAAGAGAGAGAGAGACAGGGAAAAAGAAACAGAAAGAGAGGAGAGGAGAAGTGATGAAGTAGGTGGAAAAATGTTTTCCTTGCTATTGTCTTAGAACAGAATGACTGCATTTCTGTTTCTGGTCTTCAATTTTCTCTTTCCTCATGTGGCTCATTGCCTTCATCTTCAACATCTTTAATTTTTCTTTTTACATGTTAATTTTACCCTTAATAAACATTTACATGGTTTTTCTGTTTTTCCCCCCATTTGGATATAAGTCTTCCCAGTTGTATAAAAATTTTCCATCTCATGTTCCTGTATTTCTCAAAGGGCGAATTCCACATTGGTCGCTGCAGCCCGGGGGATCCACTAGTTCTAGAGCGGCCGCANCGCNNNGCTCCNATTCGCCCTATAGTGAGTCGTATTACGCGCGCTCACTGGCCGTCGTTNANACGTCNNGACTGGGNAANCCNGGNNTTACCCNACTTAATCNNCTTGCAGCACATCCCCNTTNN

>Jurkat_Stim2_KO1_20-M13R_D03.ab1 **Same as Jurkat_STIM2_KO1_13-M13R_E02.ab1**
NNNNNNNNNNNNNNNNTTACGCNNNCGCGCNTTAACCCTCACTAAAGGGAACAAAAGCTGGGTACCGGGCCCCCCCTCGAGGTCGACGGTATCGATAAGCTTGATATCCACTGTGGAATTCGCCCTTTGAGAAATACAGGAACATGAGATGGAAAATTTTTATACAACTGGGAAGACTTATATCCAAATGGGGGGAAAAACAGAAAAACCATGTAAATGTTTATTAAGGGTAAAATTAACATGTAAAAAGAAAAATTAAAGATGTTGAAGATGAAGGCAATGAGCCACATGAGGAAAGAGAAAATTGAAGACCAGAAACAGAAATGCAGTCATTCTGTTCTAAGACAATAGCAAGGAAAACATTTTTCCACCTACTTCATCACTTCTCCTCTCCTCTCTTTCTGTTTCTTTTTCCCTGTCTCTCTCTCTCTTTTTGTTTTACTGTAATACTTCAATTCCACCATCTTTGTCATCATCCATTTGTTTATGTATTGTTTGAAGAGCTTCCAGACTAAATCTGTCTTCTTCTGTAAAGCATGGTGGACTCAGTGACATGCAGGGATCTGAAAGAGAACAAAGGGCAATTACTAACTTGCCATACCAAAACAAAGGCTATAGTTAGAGAGTATAATTTAAAAGAAAACCAACAGAATTAAACTTCAGACTAAGTTAAACTAAGAGACTTTAAAACGTCTAACAGAATATTCTTCACATCAAAAGTATAATATATGAACACAACTGACTAAAAAGAAATAATCCAATTTAANTAACATCAGGCAACTACAACCAGGAGTAAGGNGAATTCCACATTGGTCGCTGCANCCCGGGGGATCCACTAGTTCTAGAGCGGCCGCACNCGNNGCTCCAATTCGCCCTATAGTGAGTCGTATTACGCGCGCTCACTGGCCGTCGTTTNNNACGTCNNGACTGGGAAAACCCNGGCGTTACCCNACTTAANCG

>Jurkat_Stim2_KO2_21-M13R_E03.ab1 **14 nt deletion marked in red**
NNNNNNNNNNNNNNNNACGCCAGCGCGCAATTAACCCTCACTAAAGGGAACAAAAGCTGGGTACCGGGCCCCCCCTCGAGGTCGACGGTATCGATAAGCTTGATATCCACTGTGGAATTCGCCCTTACTCCTGGTTGTAGTTGCCTGATGTTAATTAAATTGGATTATTTCTTTTTAGTCAGTTGTGTTCATATATTATACTTTTGATGTGAAGAATATTCTGTTAGACGTTTTAAAGTCTCTTAGTTTAACTTAGTCTGAAGTTTAATTCTGTTGGTTTTCTTTTAAATTATACTCTCTAACTATAGCCTTTGTTTTGGTATGGCAAGTTAGTAATTGCCCTTTGTTCTCTTTCAGATCCCTGCATGTCACTGAGTCCACCATGCTTTACAGAAGAAGACAGATTTAGTCTGGAAGCTCTTCAAACAATACATAAACAAATGGATGATGACAAAGA **_ _ _ _ _ _ _ _ _ _ _ _ _ _** TAGAGGAAAGTGATGAAGTAGGTGGAAAAATGTTTTCCTTGCTATTGTCTTAGAACAGAATGACTGCATTTCTGTTTCTGGTCTTCAATTTTCTCTTTCCTCATGTGGCTCATTGCCTTCATCTTCAACATCTTTAATTTTTCTTTTTACATGTTAATTTTACCCTTAATAAACATTTACATGGTTTTTCTGTTTTTCCCCCCATTTGGATATAAGTCTTCCCAGTTGTATAAAAATTTTCCATCTCATGTTCCTGTATTTCTCAAAGGGCGAATTCCACATTGGTCGCTGCAGCCCGGGGGATNCACTAGTTCTAGAGCGGCCGCACCGCNNAGCTCCAATTCGCCCTATAGTGAGTCGTATTACGCGCGCTCACTGGCCGTCNTTTACACGTCNTGACTGGGAAANCCTNNNTTACCCAACTANCGCNNCAGCNCATCCCCTTTCGCAGCTGGNGTATANCGANAGGCCCGCANCGATTAANTTTTGNNN

>Jurkat_Stim2_KO2_22-M13R_F03.ab1 **10 nt deletion marked in red**
NNNNNNNNNNNNNTTACGCCAGCGCGCAATTAACCCTCACTAAAGGGAACAAAAGCTGGGTACCGGGCCCCCCCTCGAGGTCGACGGTATCGATAAGCTTGATATCCACTGTGGAATTCGCCCTTTGAGAAATACAGGAACATGAGATGGAAAATTTTTATACAACTGGGAAGACTTATATCCAAATGGGGGGAAAAACAGAAAAACCATGTAAATGTTTATTAAGGGTAAAATTAACATGTAAAAAGAAAAATTAAAGATGTTGAAGATGAAGGCAATGAGCCACATGAGGAAAGAGAAAATTGAAGACCAGAAACAGAAATGCAGTCATTCTGTTCTAAGACAATAGCAAGGAAAACATTTTTCCACCTACTTCATCACTTT **_ _ _ _ _ _ _ _ _ _** AATTCCACCATCTTTGTCATCATCCATTTGTTTATGTATTGTTTGAAGAGCTTCCAGACTAAATCTGTCTTCTTCTGTAAAGCATGGTGGACTCAGTGACATGCAGGGATCTGAAAGAGAACAAAGGGCAATTACTAACTTGCCATACCAAAACAAAGGCTATAGTTAGAGAGTATAATTTAAAAGAAAACCAACAGAATTAAACTTCAGACTAAGTTAAACTAAGAGACTTTAAAACGTCTAACAGAATATTCTTCACATCAAAAGTATAATATATGAACACAACTGACTAAAAAGAAATAATCCAATTTAATTAACATCANGCAACTACAACCAGGAGTNAAGGNCGAATTCCACATTGGTCGCTGCAGCCCGGGGGATCCACTAGTTCTAGAGCGGCCGCACCGCGGGAGCTCCAATTCGCCCTATAGTGAGTCGTATTACGCGCGCTCACTGGNCGTCGTTTTACANGTCNNGACTGNNAAANCCTNNNTTACCCAANNNNCGCNNCAGCACATCCCCTTTCNCAGCTGGNGTAATAGCGANNGNCNCACGATAATTTGNCATGANNTNNNCAANNNTNNTCNCNANNTCNNAANTNAANNANTTTAANNANCNN

>Jurkat_Stim2_KO2_23-M13R_G03.ab1 **Same as >Jurkat_Stim2_KO2_21-M13R_E03.ab1**
NNNNNNNNNNNNNTACGCNNNCGCGCAATTAACCCTCACTAAAGGGAACAAAAGCTGGGTACCGGGCCCCCCCTCGAGGTCGACGGTATCGATAAGCTTGATATCCACTGTGGAATTCGCCCTTACTCCTGGTTGTAGTTGCCTGATGTTAATTAAATTGGATTATTTCTTTTTAGTCAGTTGTGTTCATATATTATACTTTTGATGTGAAGAATATTCTGTTAGACGTTTTAAAGTCTCTTAGTTTAACTTAGTCTGAAGTTTAATTCTGTTGGTTTTCTTTTATATTATACTCTCTAACTATAGCCTTTGTTTTGGTATGGCAAGTTAGTAATTGCCCTTTGTTCTCTTTCAGATCCCTGCATGTCACTGAGTCCACCATGCTTTACAGAAGAAGACAGATTTAGTCTGGAAGCTCTTCAAACAATACATAAACAAATGGATGATGACAAAGATAGAGGAAAGTGATGAANTAAGTGGAAAAATGTTTTCCTTGCTAGTGTCTTANAACAAAATGACTGCATTTCTGTNTCTGGNCNGCNNTTTTCTCTTTCCTCATGTGGCTCATTGCCTTCATCTTCAACATCTTTAATTTATCTTTTTACATGTTNATTTTACNCTTAATAAACATTTACATGGTTGNTCTGTTTNTCCCCCCATTTGGATATAAATCTTCCCACTTGTATAAAAATTNNCCATCTCATGTTCCTGTATTTCTCANAGGGCGAATTCNACATTGNTCGCTGCANCCCGGGGGATCCATTANNTCTATAGCGGCCGCACCNNGGGAGCTCCATTCNCNCTATANTGAGTCNNATNACNCNCGCTCACTGNCCGTCNNTTTANANCGTCNNGNNNGANAGAACNNNGNCNNTACCCAACTTAANCNCNNTGCATCACANCCCCCCNTNCCNTCANNTGGCNNANNN

>Jurkat_Stim2_KO2_24-M13R_H03.ab1
**Vector only, result invalid**

>Jurkat_Stim2_KO2_25-M13R_A04.ab1 **Same as >Jurkat_Stim2_KO2_21-M13R_E03.ab1**
NNNNNNNNNNNNNTTACGCCAGCGCGNNNTAACCCTCACTAAAGGGAACAAAAGCTGGGTACCGGGCCCCCCCTCGAGGTCGACGGTATCGATAAGCTTGATATCCACTGTGGAATTCGCCCTTACTCCTGGTTGTAGTTGCCTGATGTTAATTAAATTGGATTATTTCTTTTTAGTCAGTTGTGTTCATATATTATACTTTTGATGTGAAGAATATTCTGTTAGACGTTTTAAAGTCTCTTAGTTTAACTTAGTCTGAAGTTTAATTCTGTTGGTTTTCTTTTATATTATACTCTCTAACTATAGCCTTTGTTTTGGTATGGCAAGTTAGTAATTGCCCTTTGTTCTCTTTCAGATCCCTGCATGTCACTGAGTCCACCATGCTTTACAGAAGAAGACAGATTTAGTCTGGAAGCTCTTCAAACAATACATAAACAAATGGATGATGACAAAGATAGAGGAAAGTGATGAAGTAGGTGGAAAAATGTTTTCCTTGCTATTGTCTTAGAACAGAATGACTGCATTTCTGTTTCTGGTCTTCAATTTTCTCTTTCCTCATGTGGCTCATTGCCTTCATCTTCAACATCTTTAATTTTTCTTTTTACATGTTAATTTTACCCTTAATAAACATTTACATGGTTTTTCTGTTTTTCCCCCCATTTGGATATAAGTCTTCCCAGTTGTATAAAAATTTTCCATCTCATGTTCCTGTATTTCTCAAAGGGCGAATTCCACATTGGTCGCTGCAGCCCGGGGGATCCACTAGTTCTAGAGCGGCCGCACCGCGGGAGCTCCAATTCGCCCTATAGTGAGTCGTATTACGCGCGCTCACTGGNCGTCGTTTACAACGTCGTGACTGGGAAACCCTGGNGTTACCCAACTTAATCGCCTTGCAGCACATCCCCTTTCGCCAGCTGGCGTAATAGCGANNANN

>Jurkat_Stim2_KO2_26-M13R_B04.ab1 **10 nt deletion marked in red**
NNNNNNNNNNNTNNTTACGCCAGCGCGCAATTAACCCTCACTAAAGGGAACAAAAGCTGGGTACCGGGCCCCCCCTCGAGGTCGACGGTATCGATAAGCTTGATATCCACTGTGGAATTCGCCCTTTGAGAAATACAGGAACATGAGATGGAAAATTTTTATACAACTGGGAAGACTTATATCCAAATGGGGGGAAAAACAGAAAAACCATGTAAATGTTTATTAAGGGTAAAATTAACATGTAAAAAGAAAAATTAAAGATGTTGAAGATGAAGGCAATGAGCCACATGAGGAAAGAGAAAATTGAAGACCAGAAACAGAAATGCAGTCATTCTGTTCTAAGACAATAGCAAGGAAAACATTTTTCCACCTACTTCATCACTTT **_ _ _ _ _ _ _ _ _ _** AATTCCACCATCTTTGTCATCATCCATTTGTTTATGTATTGTTTGAAGAGCTTCCAGACTAAATCTGTCTTCTTCTGTAAAGCATGGTGGACTCAGTGACATGCAGGGATCTGAAAGAGAACAAAGGGCAATTACTAACTTGCCATACCAAAACAAAGGCTATAGTTAGAGAGTATAATTTAAAAGAAAACCAACAGAATTAAACTTCAGACTAAGTTAAACTAAGAGACTTTAAAACGTCTAACAGAATATTCTTCACATCAAAAGTATAATATATGAACACAACTGACTAAAAAGAAATAATCCAATTTAATTAACATCANGCAACTACAACCAGGAGTAANGGCGAATTCCACATTGGTCGCTGCAGCCCGGGGGATCCACTAGTTCTAGAGCGGCCGCACCGCGGGAGCTCCAATTCGCCCTATAGTGAGTCGTATTACGCGCGCTCACTGGCCNGTCGTTTTACAACGTCNTGACTGGGAAAACCCTNNGTTACCCAACTTAATCGCCTTGCAGCACATCCCCCTTTCGCCAGCTGGCGTAATAGCGAANANNNCCGNNNCGNN

>Jurkat_Stim2_KO2_27-M13R_C04.ab1 **Same as >Jurkat_Stim2_KO2_21-M13R_E03.ab1**
NNNNNNNNCNNNNTTACGCCAGCGCGCAATTAACCCTCACTAAAGGGAACAAAAGCTGGGTACCGGGCCCCCCCTCGAGGTCGACGGTATCGATAAGCTTGATATCCACTGTGGAATTCGCCCTTACTCCTGGTTGTAGTTGCCTGATGTTAATTAAATTGGATTATTTCTTTTTAGTCAGTTGTGTTCATATATTATACTTTTGATGTGAAGAATATTCTGTTAGACGTTTTAAAGTCTCTTAGTTTAACTTAGTCTGAAGTTTAATTCTGTTGGTTTTCTTTTATATTATACTCTCTAACTATAGCCTTTGTTTTGGTATGGCAAGTTAGTAATTGCCCTTTGTTCTCTTTCAGATCCCTGCATGTCACTGAGTCCACCATGCTTTACAGAAGAAGACAGATTTAGTCTGGAAGCTCTTCAAACAATACATAAACAAATGGATGATGACAAAGATAGAGGAAAGTGATGAAGTAGGTGGAAAAATGTTTTCCTTGCTATTGTCTTAGAACAGAATGACTGCATTTCTGTTTCTGGTCTTCAATTTTCTCTTTCCTCATGTGGCTCATTGCCTTCATCTTCAACATCTTTAATTTTTCTTTTTACATGTTAATTTTACCCTTAATAAACATTTACATGGTTTTTCTGTTTTTCCCCCCATTTGGATATAAGTCTTCCCAGTTGTATAAAAATTTTCCATCTCATGTTCCTGTATTTCTCAAAGGGCGAATTCCACATTGGTCGCTGCAGCCCGGGGGATCCACTAGTTCTAGAGCGGCCGCACCGCGGGAGCTCCAATTCGCCCTATAGTGAGTCGTATTACGCGCGCTCACTGGNCNGTCGTTTTACAACGTCGTGACTGGGNAAACCCTGGCGTTACCCAACTTAATCGCCTTGCAGCACATCCCCCTTTCGCCAGCTGGCGTAATAGCGANAGGCCCNCNCGATNAATTTGGTCATGAGATTATCAAAAGNATNTCACCTANATCCTTTNAANNAAAATGAAGTTTN

>Jurkat_Stim2_KO2_28-M13R_D04.ab1 **Same as >Jurkat_Stim2_KO2_21-M13R_E03.ab1**
NNNNNNNNNNNNNNNNNACGCCNNGCGCGCNNTAACCCTCACTAAAGGGAACAAAAGCTGGGTACCGGGCCCCCCCTCGAGGTCGACGGTATCGATAAGCTTGATATCCACTGTGGAATTCGCCCTTACTCCTGGTTGTAGTTGCCTGATGTTAATTAAATTGGATTATTTCTTTTTAGTCAGTTGTGTTCATATATTATACTTTTGATGTGAAGAATATTCTGTTAGACGTTTTAAAGTCTCTTAGTTTAACTTAGTCTGAAGTTTAATTCTGTTGGTTTTCTTTTAAATTATACTCTCTAACTATAGCCTTTGTTTTGGTATGGCAAGTTAGTAATTGCCCTTTGTTCTCTTTCAGATCCCTGCATGTCACTGAGTCCACCATGCTTTACAGAAGAAGACAGATTTAGTCTGGAAGCTCTTCAAACAATACATAAACAAATGGATGATGACAAAGATAGAGGAAAGTGATGAAGTAGGTGGAAAAATGTTTTCCTTGCTATTGTCTTAGAACAGAATGACTGCATTTCTGTTTCTGGTCTTCAATTTTCTCTTTCCTCATGTGGCTCATTGCCTTCATCTTCAACATCTTTAATTTTTCTTTTTACATGTTAATTTTACCCTTAATAAACATTTACATGGTTTTTCTGTTTTTCCCCCCATTTGGATATAAGTCTTCCCAGTTGTATAAAAATTTTCCATCTCATGTTCCTGTATTTCTCAAAGGGCGAATTCCACATTGGTCGCTGCAGCCCGGGGGATCCACTAGTTCTAGAGCGGCCGCACCGCGGGAGCTCCAATTCGCCCTATAGTGAGTCGTATTACGCGCGCTCACTGGCCGTCGTTTTACAACGTCNTGACTGGGANNACCNNNNGTTACCCANCTTAATCGCCTTGCAGCACATCCCCCNTTCGCCAGCTGGCGTAATANCGAANAGGCCCGCNNCGATTAAATTTTGGTNNN

>Jurkat_Stim2_DKO_29-M13R_E04.ab1 **Same as >Jurkat_Stim2_KO2_21-M13R_E03.ab1**
NNNNNNNNNNNNNNTTACGCCAGCGCGCAATTAACCCTCACTAAAGGGAACAAAAGCTGGGTACCGGGCCCCCCCTCGAGGTCGACGGTATCGATAAGCTTGATATCCACTGTGGAATTCGCCCTTACTCCTGGTTGTAGTTGCCTGATGTTAATTAAATTGGATTATTTCTTTTTAGTCAGTTGTGTTCATATATTATACTTTTGATGTGAAGAATATTCTGTTAGACGTTTTAAAGTCTCTTAGTTTAACTTAGTCTGAAGTTTAATTCTGTTGGTTTTCTTTTAAATTATACTCTCTAACTATAGCCTTTGTTTTGGTATGGCAAGTTAGTAATTGCCCTTTGTTCTCTTTCAGATCCCTGCATGTCACTGAGTCCACCATGCTTTACAGAAGAAGACAGATTTAGTCTGGAAGCTCTTCAAACAATACATAAACAAATGGATGATGACAAAGATAGAGGAAAGTGATGAAGTAGGTGGAAAAATGTTTTCCTTGCTATTGTCTTAGAACAGAATGACTGCATTTCTGTTTCTGGTCTTCAATTTTCTCTTTCCTCATGTGGCTCATTGCCTTCATCTTCAACATCTTTAATTTTTCTTTTTACATGTTAATTTTACCCTTAATAAACATTTACATGGTTTTTCTGTTTTTCCCCCCATTTGGATATAAGTCTTCCCAGTTGTATAAAAATTTTCCATCTCATGTTCCTGTATTTCTCAAAGGGCGAATTCCACATTGGTCGCTGCAGCCCGGGGGATCCACTAGTTCTAGAGCGGCCGCACCGCGGGAGCTCCAATTCGCCCTATAGTGAGTCGTATTACGCGCGCTCACTGGNCNGTCGTTTTANANGTCNTGACTGGGAAAACCCTNNNTTACCCAACTTAATCGCCTTGCAGCACATCCCCCTTTCGCCAGCTGGCGTAATAGCGAANNANGCCCNNNCCN

>Jurkat_Stim2_DKO_30-M13R_F04.ab1 **Same as >Jurkat_Stim2_KO2_22-M13R_F03.ab1**
NNNNNNNNNNNTTACGCCAGCGCGCAATTAACCCTCACTAAAGGGAACAAAAGCTGGGTACCGGGCCCCCCCTCGAGGTCGACGGTATCGATAAGCTTGATATCCACTGTGGAATTCGCCCTTACTCCTGGTTGTAGTTGCCTGATGTTAATTAAATTGGATTATTTCTTTTTAGTCAGTTGTGTTCATATATTATACTTTTGATGTGAAGAATATTCTGTTAGACGTTTTAAAGTCTCTTAGTTTAACTTAGTCTGAAGTTTAATTCTGTTGGTTTTCTTTTATATTATACTCTCTAACTATAGCCTTTGTTTTGGTATGGCAAGTTAGTAATTGCCCTTTGTTCTCTTTCAGATCCCTGCATGTCACTGAGTCCACCATGCTTTACAGAAGAAGACAGATTTAGTCTGGAAGCTCTTCAAACAATACATAAACAAATGGATGATGACAAAGATGGTGGAATTAAAGTGATGAAGTAGGTGGAAAAATGTTTTCCTTGCTATTGTCTTAGAACAGAATGACTGCATTTCTGTTTCTGGTCTTCAATTTTCTCTTTCCTCATGTGGCTCATTGCCTTCATCTTCAACATCTTTAATTTTTCTTTTTACATGTTAATTTTACCCTTAATAAACATTTACATGGTTTTTCTGTTTTTCCCCCCATTTGGATATAAGTCTTCCCAGTTGTATAAAAATTTTCCATCTCATGTTCCTGTATTTCTCAAAGGGCGAATTCCACATTGGTCGCTGCAGCCCGGGGGATCCACTAGTTCTAGAGCGGCCGCACCGCGGGAGCTCCAATTCGCCCTATAGTGAGTCGTATTACGCGCGCTCACTGGNNGTCGTTTTACANGTCNTGACTGGGAAAACCCTGGNGTTACCCACTTANCGCNTGCAGCACATCCCCCTTTCNCAGCTGGCGTANANCNANANGCCCGCACCGATTAANTTTTNGTCATGAGATTATCAAAAAGGANNNTNNNCN

>Jurkat_Stim2_DKO_31-M13R_G04.ab1 **Same as >Jurkat_Stim2_KO2_21-M13R_E03.ab1**
NNNNNNNCNTNNTTACGCCAGCGCGCAATTAACCCTCACTAAAGGGAACAAAAGCTGGGTACCGGGCCCCCCCTCGAGGTCGACGGTATCGATAAGCTTGATATCCACTGTGGAATTCGCCCTTACTCCTGGTTGTAGTTGCCTGATGTTAATTAAATTGGATTATTTCTTTTTAGTCAGTTGTGTTCATATATTATACTTTTGATGTGAAGAATATTCTGTTAGACGTTTTAAAGTCTCTTAGTTTAACTTAGTCTGAAGTTTAATTCTGTTGGTTTTCTTTTATATTATACTCTCTAACTATAGCCTTTGTTTTGGTATGGCAAGTTAGTAATTGCCCTTTGTTCTCTTTCAGATCCCTGCATGTCACTGAGTCCACCATGCTTTACAGAAGAAGACAGATTTAGTCTGGAAGCTCTTCAAACAATACATAAACAAATGGATGATGACAAAGATAGAGGAAAGTGATGAAGTAAGTGGAAAAATGTTTTCCTTGCTATTGNCTTACAACAAAATGACTGCATTTCTGTTTCTGGTCNTCNNTTTTCTCTTTCCTCATGTGGCTCATTGCCTTCATCTTCAACATCTTTAATTTTTCTTTTTACATGTTAATTTTACNCTTAATAAACATTTACATGGNTGTTCTGTNTNTCCCCCCATTTGGATATAANTCTTCCCACTTGTATAAAAATTNTCTATCTCATGTTCCTGTATTTCTCANANGGCGAATTCCATATTGGTCGCTGCANCCCGGGGGGATCCATTANTTCTATAGCGGCCGCNCCGCCGNGAGCTCNATTCNNNCTATANTGAGTCNNATTACNCGCGCTCACTGNCCGTCGTTTACANCGTCNNGNCTGNNANAACNCTNNNTTACCTAACTTAANCGACCTTGCATCNNATCCCCCCNTNNCNTCAGCTGGCNNNANNNCNAANANGCCNNCNNCGATTAANTNNNN

>Jurkat_Stim2_DKO_32-M13R_H04.ab1 **Same as >Jurkat_Stim2_KO2_21-M13R_E03.ab1**
NNNNNNNNNNNNTTACGCCAGCGCGCAATTAACCCTCACTAAAGGGAACAAAAGCTGGGTACCGGGCCCCCCCTCGAGGTCGACGGTATCGATAAGCTTGATATCCACTGTGGAATTCGCCCTTTGAGAAATACAGGAACATGAGATGGAAAATTTTTATACAACTGGGAAGACTTATATCCAAATGGGGGGAAAAACAGAAAAACCATGTAAATGTTTATTAAGGGTAAAATTAACATGTAAAAAGAAAAATTAAAGATGTTGAAGATGAAGGCAATGAGCCACATGAGGAAAGAGAAAATTGAAGACCAGAAACAGAAATGCAGTCATTCTGTTCTAAGACAATAGCAAGGAAAACATTTTTCCACCTACTTCATCACTTTCCTCTATCTTTGTCATCATCCATTTGTTTATGTATTGTTTGAAGAGCTTCCAGACTAAATCTGTCTTCTTCTGTAAAGCATGGTGGACTCAGTGACATGCAGGGATCTGAAAGAGAACAAAGGGCAATTACTAACTTGCCATACCAAAACAAAGGCTATAGTTAGAGAGTATAATTTAAAAGAAAACCAACAGAATTAAACTTCAGACTAAGTTAAACTAAGAGACTTTAAAACGTCTAACAGAATATTCTTCACATCAAAAGTATAATATATGAACACAACTGACTAAAAAGAAATAATCCAATTTAATTAACATCNNCAACTACAACCAGGAGTAANGGCGAATTCCACATTGGTCGCTGCAGCCCGGGGGGATCCACTAGTTCTAGAGCGGCCGCACCGCGGGAGCTCCAATTCGCCCTATAGTGAGTCGTATTANGCGCGCTCACTGGNCGTCGTTTACNACGTCNNGACTGGNNNAACCCTGNCGTTACCCAACTTAANCGCCTNGCANCACATCCCCCTTTCGTCAGCTGCNTNNNANCGAANAGGCCNGNCACCGANNAATTNNNNNTCANNGANNNNATNN

>Jurkat_Stim2_DKO_33-M13R_A05.ab1 **Same as >Jurkat_Stim2_KO2_21-M13R_E03.ab1**
NNNNNNNNNNNNNNNNNTACGCCAAGCGCGCAATTAACCCTCACTAAAGGGAACAAAAGCTGGGTACCGGGCCCCCCCTCGAGGTCGACGGTATCGATAAGCTTGATATCCACTGTGGAATTCGCCCTTTGAGAAATACAGGAACATGAGATGGAAAATTTTTATACAACTGGGAAGACTTATATCCAAATGGGGGGAAAAACAGAAAAACCATGTAAATGTTTATTAAGGGTAAAATTAACATGTAAAAAGAAAAATTAAAGATGTTGAAGATGAAGGCAATGAGCCACATGAGGAAAGAGAAAATTGAAGACCAGAAACAGAAATGCAGTCATTCTGTTCTAAGACAATAGCAAGGAAAACATTTTTCCACCTACTTCATCACTTTCCTCTATCTTTGTCATCATCCATTTGTTTATGTATTGTTTGAAGAGCTTCCAGACTAAATCTGTCTTCTTCTGTAAAGCATGGTGGACTCAGTGACATGCAGGGATCTGAAAGAGAACAAAGGGCAATTACTAACTTGCCATACCAAAACAAAGGCTATAGTTAGAGAGTATAATTTAAAAGAAAACCAACAGAATTAAACTTCAGACTAAGTTAAACTAAGAGACTTTAAAACGTCTAACAGAATATTCTTCACATCAAAAGTATAATATATGAACACAACTGACTAAAAAGAAATAATCCAATTTAATTAACATCANGCAACTACAACCAGGAGTAANGGCGAATTCCACATTGGTCGCTGCAGCCCGGGGGATCCACTAGTTCTAGAGCGGCCGCACCGCGGGAGCTCCAATTCGCCCTATAGTGAGTCGTATTACGCGCGCTCACTGGCCGTCGTTTACACGTCNNGACTGGGAAAACCCTNNNTTACCCAACTTAATCGNCNTGCAGCACATCCCCCTTTCGCAGCTGGNGTAATANCNNANAGNCCGCANCGATTAAATTTTNGTCATGANANTNNCAAAANGATNTCNCCTAGATCCTTTAAATNAAAATGAAGTTTA

>Jurkat_Stim2_DKO_34-M13R_C05.ab1 **Same as >Jurkat_Stim2_KO2_22-M13R_F03.ab1**
NNNNNNNNNNNNNTTACGCCAAGCGCGCAATTAACCCTCACTAAAGGGAACAAAAGCTGGGTACCGGGCCCCCCCTCGAGGTCGACGGTATCGATAAGCTTGATATCCACTGTGGAATTCGCCCTTTGAGAAATACAGGAACATGAGATGGAAAATTTTTATACAACTGGGAAGACTTATATCCAAATGGGGGGAAAAACAGAAAAACCATGTAAATGTTTATTAAGGGTAAAATTAACATGTAAAAAGAAAAATTAAAGATGTTGAAGATGAAGGCAATGAGCCACATGAGGAAAGAGAAAATTGAAGACCAGAAACAGAAATGCAGTCATTCTGTTCTAAGACAATAGCAAGGAAAACATTTTTCCACCTACTTCATCACTTCCATTCCACCNTCTTTGCCATCATCCGTTTGTTTATGGATTGATTGAATAGCTTCCAAACTAAATCTGTCTTCTTCTGTAAAGCATGGTGGACTCACTGACATGGATGGATCTGAAAGANAACGAAGGGTAATTACTAACTTGCCNTACCNAAACGCAGGCTATAGTTAGAGAGTATNATATNAAAGANAACCAACATAATTANACNNCNNACTAANTTAAACTANACATACTTTAAAACGTCTAANANANTATTCTTCACATCATANGTATAATATATGAACACGACTGACTAAAAAGAANTANNNNNNNTTAATTAANNTCNNGCACCTACAACNAGNANNNNNGNCTAATTCCTGNTCGGTCNCTGCNNNGNGNGNGATCCACTANTTCTAGAGCNGCCCGCNCNGNGNGAGCACNNANTCNCCCTATAGTGAGTCNTATTACGCGCGCTGACTGGTCGTNNTTNTANNCGTGNNTGACTGGNAAAANGNCTGTCGTTAACCTANTTAANCNNCTTGCANCNCNNCNNNCTNNNGCNGCNGGNNTAANTAANNANNNNGCCCCCNNCCNANTN

>Jurkat_Stim2_DKO_35-M13R_E05.ab1 **Same as >Jurkat_Stim2_KO2_26-M13R_B04.ab1**
NNNNNNNNNNNNNNNNTACGCCAGCGCGCAATTAACCCTCACTAAAGGGAACAAAAGCTGGGTACCGGGCCCCCCCTCGAGGTCGACGGTATCGATAAGCTTGATATCCACTGTGGAATTCGCCCTTACTCCTGGTTGTAGTTGCCTGATGTTAATTAAATTGGATTATTTCTTTTTAGTCAGTTGTGTTCATATATTATACTTTTGATGTGAAGAATATTCTGTTAGACGTTTTAAAGTCTCTTAGTTTAACTTAGTCTGAAGTTTAATTCTGTTGGTTTTCTTTTAAATTATACTCTCTAACTATAGCCTTTGTTTTGGTATGGCAAGTTAGTAATTGCCCTTTGTTCTCTTTCAGATCCCTGCATGTCACTGAGTCCACCATGCTTTACAGAAGAAGACAGATTTAGTCTGGAAGCTCTTCAAACAATACATAAACAAATGGATGATGACAAAGATGGTGGAATTAAAGTGATGAAGTAGGTGGAAAAATGTTTTCCTTGCTATTGTCTTAGAACAGAATGACTGCATTTCTGTTTCTGGTCTTCAATTTTCTCTTTCCTCATGTGGCTCATTGCCTTCATCTTCAACATCTTTAATTTTTCTTTTTACATGTTAATTTTACCCTTAATAAACATTTACATGGTTTTTCTGTTTTTCCCCCCATTTGGATATAAGTCTTCCCAGTTGTATAAAAATTTTCCATCTCATGTTCCTGTATTTCTCAAAGGGCGAATTCCACATTGGTCGCTGCAGCCCGGGGGATCCACTAGTTCTAGAGCGGCCGCACCGCGGGAGCTCCAATTCGCCCTATAGTGAGTCGTATTACGCGCGCTCACTGGNNGTCGTTTTACAACGTCGTGACTGGGAAAACCCNGGNGTTACCCAACTTAATCGCCTTGCAGCACATCCCCCTTTCGCCAGCTGGCGTAATAGCGAANAGGCCCNNNANCGANN

>Jurkat_Stim2_DKO_36-M13R_F05.ab1 **Same as >Jurkat_Stim2_KO2_26-M13R_B04.ab1**
NNNNNNNNNNNNNTTACGCCAGCGCGCAATTAACCCTCACTAAAGGGAACAAAAGCTGGGTACCGGGCCCCCCCTCGAGGTCGACGGTATCGATAAGCTTGATATCCACTGTGGAATTCGCCCTTACTCCTGGTTGTAGTTGCCTGATGTTAATTAAATTGGATTATTTCTTTTTAGTCAGTTGTGTTCATATATTATACTTTTGATGTGAAGAATATTCTGTTAGACGTTTTAAAGTCTCTTAGTTTAACTTAGTCTGAAGTTTAATTCTGTTGGTTTTCTTTTATATTATACTCTCTAACTATAGCCTTTGTTTTGGTATGGCAAGTTAGTAATTGCCCTTTGTTCTCTTTCAGATCCCTGCATGTCACTGAGTCCACCATGCTTTACAGAAGAAGACAGATTTAGTCTGGAAGCTCTTCAAACAATACATAAACAAATGGATGATGACAAAGATGGTGGAATTAAAGTGATGAAGTAGGTGGAAAAATGTTTTCCTTGCTATTGTCTTAGAACAGAATGACTGCATTTCTGTTTCTGGTCTTCAATTTTCTCTTTCCTCATGTGGCTCATTGCCTTCATCTTCAACATCTTTAATTTTTCTTTTTACATGTTAATTTTACCCTTAATAAACATTTACATGGTTTTTCTGTTTTTCCCCCCATTTGGATATAAGTCTTCCCAGTTGTATAAAAATTTTCCATCTCATGTTCCTGTATTTCTCAAAGGGCGAATTCCACATTGGTCGCTGCAGCCCGGGGGATCCACTAGTTCTAGAGCGGCCGCACCGCGGGAGCTCCAATTCGCCCTATAGTGAGTCGTATTACGCGCGCTCACTGGNCGTCGTTTACACGTCGTGACTGGGAAAACCCTGGNGTTACCCAACTTAATCGCCTTGCAGCACATCCCCCTTTCGCCAGCTGGCGTAATAGCGAANNAGGCCCNNCNNNNNNTTA

>Jurkat_S1DKO_1-M13R_A11.ab1 **Mutation marked in blue, 17 nt deletion marked in red**
NNNNNNNNNNNNNNNNTACGCCAGCGCGCNTTAACCCTCACTAAAGGGAACAAAAGCTGGGTACCGGGCCCCCCCTCGAGGTCGACGGTATCGATAAGCTTGATATCCACTGTGGAATTCGCCCTTAAGAAAGGGGCTGCCTCAAGAGACCAGCAAGCTCCCTATCTCTGGAGATAAAGCAGCAGAACCTGGAAGATTCCTATGCCAGGGTTAGGTGGGTGA**G**GAAAGGGGTAGGAATAGGATAGAATTCAAAAAGTATCCTAATGAAGAAAAACGCCAACTCTGGAGAACCTTCCCCCATGCCCAGCAGAGTGACTATATTCAGTCTTTTTTAGTGACTATATGTAGAATAACTTCAATAATTTGCCCTTTACTAAGCATTTTATATACATGTTCTTCTAAGGCCAAGTTGCTGCTTCTCTCTTCTCTAAACCTTCCCACAACTAGGATTGATGACCAGGCTGCCTATTACCTTCTGATGACTTCCATGCCTTCCACAGGTCCTCCACGCTGATGAGCTTATC **_ _ _ _ _ _ _ _ _ _ _ _ _ _ _ _ _** TGTGTTTCACTGTTGGGTCATGGTAATTGAGGTCTTCCCTCANGAACTGCAAGGGAAAAGTACACAAGTCACAAGAGATACCTAGGAGTCAGCCCGTCAAGATCTCCACACAGGTCACCTGCCTCTAGCTAGCCATAACACATTCCATCTACCCAACATACAAGTGTATCACATCACTCATGTATCCTTCCTTATGCACATGTCTTANTTGCCAACTAAACTATCACTTCCTCTTTGTTACATGTCTCACTTGAAAGGGCGAATTCCACATTGGTCGCTGCAGCCCGGGGGATCCACTAGTTCTAGAGCGGCCGCACCGCGGNGCTCCAATTCGCCCTATAGTGAGTCGTATTACGCNCGCTCACTGGCCGTCGTTTTACAACGTCGTGACTGGGAAAACNNN

>Jurkat_S1DKO_2-M13R_B11.ab1 **Same as >Jurkat_S1DKO_1-M13R_A11.ab1**
NNNNNNNNNNNNNNNNNNTACGCCNAGCGCGCATTAACCCTCACTAAAGGGAACAAAAGCTGGGTACCGGGCCCCCCCTCGAGGTCGACGGTATCGATAAGCTTGATATCCACTGTGGAATTCGCCCTTAAGAAAGGGGCTGCCTCAAGAGACCAGCAAGCTCCCTATCTCTGGAGATAAAGCAGCAGAACCTGGAAGATTCCTATGCCAGGGTTAGGTGGGTGA**G**GAAAGGGGTAGGAATAGGATAGAATTCAAAAAGTATCCTAATGAAGAAAAACGCCAACTCTGGAGAACCTTCCCCCATGCCCAGCAGAGTGACTATATTCAGTCTTTTTTAGTGACTATATGTAGAATAACTTCAATAATTTGCCCTTTACTAAGCATTTTATATACATGTTCTTCTAAGGCCAAGTTGCTGCTTCTCTCTTCTCTAAACCTTCCCACAACTAGGATTGNNGACCAGGCTGCCTATTACCTTCTGATGACTTCCATGCCTTCCACAGGTCCTCCACGCTGATGAGCTTATC **_ _ _ _ _ _ _ _ _ _ _ _ _ _ _ _ _** TGTGTTTCACTGTTGGGTCATGGTAATTGAGGTCTTCCCTCANGAACTGCAAGGGAAAAGTACACAAGTCACAAGAGATACCTAGGAGTCAGCCCGTCAAGATCTCCACACAGGTCACCTGCCTCTAGCTAGCCATAACACATTCCATCTACCCAACATACAAGTGTATCACATCACTCATGTATCCTTCCTTATGCACATGTCTTANTTGCCAACTAAACTATCACTTCCTCTTTGTTACATGTCTCACTTGAAANGGCGAATTCCACATTGGTCGCTGCAGCCCGGGGGATCCACTAGTTCTAGAGCGGCCGCACCGCGGGAGCTCCAATTCGCCCTATAGTGAGTCGTANTACGCGCGCTCACTGGCCGTCGTTTACAACGTCNTGACTGGAAAACCCTGGN

>Jurkat_S1DKO_3-M13R_C11.ab1 **Mutation marked in blue, insertion marked in red**
NNNNNNNNNNNNNNNNNACNCCNAGCGCGCATTAACCCTCACTAAAGGGAACAAAAGCTGGGTACCGGGCCCCCCCTCGAGGTCGACGGTATCGATAAGCTTGATATCCACTGTGGAATTCGCCCTTAAGAAAGGGGCTGCCTCAAGAGACCAGCAAGCTCCCTATCTCTGGAGATAAAGCAGCAGAACCTGGAAGATTCCTATGCCAGGGTTAGGTGGGTGA**G**GAAAGGGGTAGGAATAGGATAGAATTCAAAAAGTATCCTAATGAAGAAAAACGCCAACTCTGGAGAACCTTCCCCCATGCCCAGCAGAGTGACTATATTCAGTCTTTTTTAGTGACTATATGTAGAATAACTTCAATAATTTGCCCTTTACTAAGCATTTTATATACATGTTCTTCTAAGGCCAAGTTGCTGCTTCTCTCTTCTCTAAACCTTCCCACAACTAGGATTGATGACCAGGCTGCCTATTACCTTCTGATGACTTCCATGCCTTCCACAGGTCCTCCACGCTGATGAGCTTATCCTCA**A**CCATGGAAGGTGCTGTGTTTCACTGTTGGGTCATGGTAATTGAGGTCTTCCCTCANGAACTGCAAGGGAAAAGTACACAAGTCACAAGAGATACCTAGGAGTCAGCCCGTCAAGATCTCCACACAGGTCACCTGCCTCTAGCTAGCCATAACACATTCCATCTACCCAACATACAAGTGTATCACATCACTCATGTATCCTTCCTTATGCACATGTCTTANTTGCCAACTAAACTATCACTTCCTCTTTGTTACATGTCTCACTGANGGCGAATTCCACATTGGTCGCTGCAGCCCGGGGGATCCACTAGTTCTAGAGCGGCCGCACCGCGGGAGCTCCAATTCGCCCTATAGTGAGTCGTANTACGCGCGCTCACTGGCCGTCGTTTTAN

>Jurkat_S1DKO_4-M13R_D11.ab1 **Same as >Jurkat_S1DKO_3-M13R_C11.ab1**
NNNNNNNNNNNCNNNNATTACGCCAGCGCGCANTANCCCTCACTAAAGGGAACAAAAGCTGGGTACCGGGCCCCCCCTCGAGGTCGACGGTATCGATAAGCTTGATATCCACTGTGGAATTCGCCCTTAAGAAAGGGGCTGCCTCAAGAGACCAGCAAGCTCCCTATCTCTGGAGATAAAGCAGCAGAACCTGGAAGATTCCTATGCCAGGGTTAGGTGGGTGA**G**GAAAGGGGTAGGAATAGGATAGAATTCAAAAAGTATCCTAATGAAGAAAAACGCCAACTCTGGAGAACCTTCCCCCATGCCCAGCAGAGTGACTATATTCAGTCTTTTTTAGTGACTATATGTAGAATAACTTCAATAATTTGCCCTTTACTAAGCATTTTATATACATGTTCTTCTAAGGCCAAGTTGCTGCTTCTCTCTTCTCTAAACCTTCCCACAACTAGGATTGATGACCAGGCTGCCTATTACCTTCTGATGACTTCCATGCCTTCCACAGGTCCTCCACGCTGATGAGCTTATCCTCA**A**CCATGGAAGGTGCTGTGTTTCACTGTTGGGTCATGGTAATTGAGGTCTTCCCTCAGGAACTGCAAGGGAAAAGTACACAAGTCACAAGAGATACCTAGGAGTCAGCCCGTCAAGATCTCCACACAGGTCACCTGCCTCTAGCTAGCCATAACACATTCCATCTACCCAACATACAAGTGTATCACATCACTCATGTATCCTTCCTTATGCACATGTCTTANTTGCCAACTAAACTATCACTTCCTCTTTGTTACATGTCTCACTTGAAAGGNCGAATTCCACATTGGTCGCTGCAGCCCGGGGGATCCACTANTTCTAGAGCGGCCGCACCGCGGGAGCTCCAATTCGCCCTATAGTGAGTCGTATTACGCGCGCTCACTGGCCG

>Jurkat_S1DKO_5-M13R_E11.ab1 **Mutation marked in blue, insertion marked in red**
NNNNNNNNNNNNNNNNNACGCCAGCGCGCATTAACCCTCACTAAAGGGAACAAAAGCTGGGTACCGGGCCCCCCCTCGAGGTCGACGGTATCGATAAGCTTGATATCCACTGTGGAATTCGCCCTTTCAAGTGAGACATGTAACAAAGAGGAAGTGATAGTTTAGTTGGCAACTAAGACATGTGCATAAGGAAGGATACATGAGTGATGTGATACACTTGTATGTTGGGTAGATGGAATGTGTTATGGCTAGCTAGAGGCAGGTGACCTGTGTGGAGATCTTGACGGGCTGACTCCTAGGTATCTCTTGTGACTTGTGTACTTTTCCCTTGCAGTTCCTGAGGGAAGACCTCAATTACCATGACCCAACAGTGAAACACAGCACCTTCCATGG**T**TGAGGATAAGCTCATCAGCGTGGAGGACCTGTGGAAGGCATGGAAGTCATCAGAAGGTAATAGGCAGCCTGGTCATCAATCCTAGTTGTGGGAAGGTTTAGAGAAGAGAGAAGCAGCAACTTGGCCTTAGAAGAACATGTATATAAAATGCTTAGTAAAGGGCAAATTATTGAAGTTATTCTACATATAGTCACTAAAAAAGACTGAATATAGTCACTCTGCTGGGCATGGGGGAAGGTTCTCCAGAGTTGGCGTTTTTCTTCATTAGGATACTTTTTGAATTCTATCCTATTCCTACCCCTTTC**C**TCACCCACCTAACCCTGGCATAGGAATCTTCCAGGTTCTGCTGCTTTATCTCCAGAGATAGGGAGCTTGCTGGTCTCTTGAGGCAGCCCCTTTCTTAAGGGCGAATTCCACATTGGTCGCTGCAGCCCGGGGGATCCACTAGTTCTAGAGCGGCCGCACCGCGGGAGCTCCAATTCGCCCTATAGTGAGTCGTATTACGCGCGCTCACTGGCCGTCN

>Jurkat_S1DKO_6-M13R_F11.ab1 **Same as >Jurkat_S1DKO_1-M13R_A11.ab1**
NNNNNNNNNNNNNTNACGCCAGCGCGNNTTAACCCTCACTAAAGGGAACAAAAGCTGGGTACCGGGCCCCCCCTCGAGGTCGACGGTATCGATAAGCTTGATATCCACTGTGGAATTCGCCCTTAAGAAAGGGGCTGCCTCAAGAGACCAGCAAGCTCCCTATCTCTGGAGATAAAGCAGCAGAACCTGGAAGATTCCTATGCCAGGGTTAGGTGGGTGA**G**GAAAGGGGTAGGAATAGGATAGAATTCAAAAAGTATCCTAATGAAGAAAAACGCCAACTCTGGAGAACCTTCCCCCATGCCCAGCAGAGTGACTATATTCAGTCTTTTTTAGTGACTATATGTAGAATAACTTCAATAATTTGCCCTTTACTAAGCATTTTATATACATGTTCTTCTAAGGCCAAGTTGCTGCTTCTCTCTTCTCTAAACCTTCCCACAACTAGGATTGATGACCAGGCTGCCTATTACCTTCTGATGACTTCCATGCCTTCCACAGGTCCTCCACGCTGATGAGCTTATC **_ _ _ _ _ _ _ _ _ _ _ _ _ _ _ _ _** TGTGTTTCACTGTTGGGTCATGGTAATTGAGGTCTTCCCTCAGGAACTGCAAGGGAAAAGTACACAAGTCACAAGAGATACCTAGGAGTCAGCCCGTCAAGATCTCCACACAGGTCACCTGCCTCTAGCTAGCCATAACACATTCCATCTACCCAACATACAAGTGTATCACATCACTCATGTATCCTTCCTTATGCACATGTCTTAGTTGCCAACTAAACTATCACTTCCTCTTTGTTACATGTCTCACTTGAAAGGGCGAATTCCACATTGGTCGCTGCANCCCGGGGGATCCACTAGTTCTAGAGCGGCCGCACCGCGGGAGCTCCAATTCNCCCTATAGTGAGTCGTATTACGCGCGCTCACTGGCCGTCGTTTTACAACGTCNTGACTGGNAAAACCCTGGNGTT

>Jurkat_S1DKO_7-M13R_G11.ab1 **Mutation marked in blue, 92 nt deletion marked in red**
NNNNNNNNNNNNNNTTACGCCAGCGCGCNNTTAACCCTCACTAAAGGGAACAAAAGCTGGGTACCGGGCCCCCCCTCGAGGTCGACGGTATCGATAAGCTTGATATCCACTGTGGAATTCGCCCTTAAGAAAGGGGCTGCCTCAAGAGACCAGCAAGCTCCCTATCTCTGGAGATAAAGCAGCAGAACCTGGAAGATTCCTATGCCAGGGTTAGGTGGGTGA**G**GAAAGGGGTAGGAATAGGATAGAATTCAAAAAGTATCCTAATGAAGAAAAACGCCAACTCTGGAGAACCTTCCCCCATGCCCAGCAGAGTGACTATATTCAGTCTTTTTTAGTGACTATATGTAGAATAACTTCAATAATTTGCCCTTTACTAAGCATTTTATATACATGTTCTTCTAAGGCCAAGTTGCTGCTTCTCTCTTCTCTAAACCTTCCCACAACTAGGATTGATGACCAGGCTGCCTATTACCTTCTGATGTCTTCCCTCAGGAA **_ _ _ _ _ _ _ _ _ _ _ _ _ _ _ _ _ _ _ _ _ _ _ _ _ _ _ _ _ _ _ _ _ _ _ _ _ _ _ _ _ _ _ _ _ _ _ _ _ _ _ _ _ _ _ _ _ _ _ _ _ _ _ _ _ __ _ _ _ _ _ _ _ _ _ _ _ _ _ _ _ _ _ _ _ _ _ _ _ _ _ _** CTGCAAGGGAAAAGTACACAAGTCACAAGAGATACCTAGGAGTCAGCCCGTCAAGATCTCCACACAGGTCACCTGCCTCTAGCTAGCCATAACACATTCCATCTACCCAACATACAAGTGTATCACATCACTCATGTATCCTTCCTTATGCACATGTCTTAGTTGCCAACTAAACTATCACTTCCTCTTTGTTACATGTCTCACTTGAAAGGGCGAATTCCACATTGGTCGCTGCAGCCCGGGGGATCCACTAGTTCTAGAGCGGCCGCACCGCGGGAGCTCCAATTCGCCCTATAGTGAGTCGTATTACGCGCGCTCACTGGCCGTCGTTTTACAACGTCNTGACTGGGAAAACCCTGGCGTTAN

>Jurkat_S1DKO_8-M13R_H11.ab1 **Same as >Jurkat_S1DKO_3-M13R_C11.ab1**
NNNNNNNNNNNNNNNNACGCNNNCGCGCANTTAACCCTCACTAAAGGGAACAAAAGCTGGGTACCGGGCCCCCCCTCGAGGTCGACGGTATCGATAAGCTTGATATCCACTGTGGAATTCGCCCTTAAGAAAGGGGCTGCCTCAAGAGACCAGCAAGCTCCCTATCTCTGGAGATAAAGCAGCAGAACCTGGAAGATTCCTATGCCAGGGTTAGGTGGGTGA**G**GAAAGGGGTAGGAATAGGATAGAATTCAAAAAGTATCCTAATGAAGAAAAACGCCAACTCTGGAGAACCTTCCCCCATGCCCAGCAGAGTGACTATATTCAGTCTTTTTTAGTGACTATATGTAGAATAACTTCAATAATTTGCCCTTTACTAAGCATTTTATATACATGTTCTTCTAAGGCCAAGTTGCTGCTTCTCTCTTCTCTAAACCTTCCCACAACTAGGATTGATGACCAGGCTGCCTATTACCTTCTGATGACTTCCATGCCTTCCACAGGTCCTCCACGCTGATGAGCTTATCCTCA**A**CCATGGAAGGTGCTGTGTTTCACTGTTGGGTCATGGTAATTGAGGTCTTCCCTCAGGAACTGCAAGGGAAAAGTACACAAGTCACAAGAGATACCTAGGAGTCAGCCCGTCAAGATCTCCACACAGGTCACCTGCCTCTAGCTAGCCATAACACATTCCATCTACCCAACATACAAGTGTATCACATCACTCATGTATCCTTCCTTATGCACATGTCTTAGTTGCCAACTAAACTATCACTTCCTCTTTGTTACATGTCTCACTTGAAAGGGCGAATTCCACATTGGTCGCTGCAGCCCGGGGGATCCACTAGTTCTAGAGCGGCCGCACCGCGGGAGCTCCAATTCGCCCTATAGTGAGTCGTATTACGCGCGCTCACTGGCCGTCGTTTTACAACGTCGTGAC

>Jurkat_S1DKO_9-M13R_A12.ab1 **Same as >Jurkat_S1DKO_3-M13R_C11.ab1**
NNNNNNNNNNNNNNNNNTACGCCAGCGCGCATTAACCCTCACTAAAGGGAACAAAAGCTGGGTACCGGGCCCCCCCTCGAGGTCGACGGTATCGATAAGCTTGATATCCACTGTGGAATTCGCCCTTAAGAAAGGGGCTGCCTCAAGAGACCAGCAAGCTCCCTATCTCTGGAGATAAAGCAGCAGAACCTGGAAGATTCCTATGCCAGGGTTAGGTGGGTGA**G**GAAAGGGGTAGGAATAGGATAGAATTCAAAAAGTATCCTAATGAAGAAAAACGCCAACTCTGGAGAACCTTCCCCCATGCCCAGCAGAGTGACTATATTCAGTCTTTTTTAGTGACTATATGTAGAATAACTTCAATAATTTGCCCTTTACTAAGCATTTTATATACATGTTCTTCTAAGGCCAAGTTGCTGCTTCTCTCTTCTCTAAACCTTCCCACAACTAGGATTGATGACCAGGCTGCCTATTACCTTCTGATGACTTCCATGCCTTCCACAGGTCCTCCACGCTGATGAGCTTATCCTCA**A**CCATGGAAGGTGCTGTGTTTCACTGTTGGGTCATGGTAATTGAGGTCTTCCCTCAGGAACTGCAAGGGAAAAGTACACAAGTCACAAGAGATACCTAGGAGTCAGCCCGTCAAGATCTCCACACAGGTCACCTGCCTCTAGCTAGCCATAACACATTCCATCTACCCAACATACAAGTGTATCACATCACTCATGTATCCTTCCTTATGCACATGTCTTAGTTGCCAACTAAACTATCACTTCCTCTTTGTTACATGTCTCACTTGAAAGGGCGAATTCCACATTGGTCGCTGCAGCCCGGGGGATCCACTAGTTCTAGAGCGGCCGCACCGCGGGAGCTNCN

>Jurkat_S1DKO_10-M13R_B12.ab1 **Same as >Jurkat_S1DKO_5-M13R_E11.ab1**
NNNNNNNNNNNCNNNNTNNNNCCAGCGCGCATTAACCCTCACTAAAGGGAACAAAAGCTGGGTACCGGGCCCCCCCTCGAGGTCGACGGTATCGATAAGCTTGATATCCACTGTGGAATTCGCCCTTCAAGTGAGACATGTAACAAAGAGGAAGTGATAGTTTAGTTGGCAACTAAGACATGTGCATAAGGAAGGATACATGAGTGATGTGATACACTTGTATGTTGGGTAGATGGAATGTGTTATGGCTAGCTAGAGGCAGGTGACCTGTGTGGAGATCTTGACGGGCTGACTCCTAGGTATCTCTTGTGACTTGTGTACTTTTCCCTTGCAGTTCCTGAGGGAAGACCTCAATTACCATGACCCAACAGTGAAACACAGCACCTTCCATGG**T**TGAGGATAAGCTCATCAGCGTGGAGGACCTGTGGAAGGCATGGAAGTCATCAGAAGGTAATAGGCAGCCTGGTCATCAATCCTAGTTGTGGGAAGGTTTAGAGAAGAGAGAAGCAGCAACTTGGCCTTAGAAGAACATGTATATAAAATGCTTAGTAAAGGGCAAATTATTGAAGTTATTCTACATATAGTCACTAAAAAAGACTGAATATAGTCACTCTGCTGGGCATGGGGGAAGGTTCTCCAGAGTTGGCGTTTTTCTTCATTAGGATACTTTTTGAATTCTATCCTATTCCTACCCCTTTC**C**TCACCCACCTAACCCTGGCATAGGAATCTTCCAGGTTCTGCTGCTTTATCTCCAGAGATAGGGAGCTTGCTGGTCTCTTGAGGCAGCCCCTTTCTTANGGNCGAATTCCACATTGGTCGCTGCAGCCCGGGGGATCCACTAGTTCTAGAGCGGCCGCACCGCGGGAGCTCCAATTCGCCCTATAGTGAGTCGTATTACGCGCGCTCAC
